# Ameliorative Effects of Berberine Against Acetamiprid-Induced Toxicity in the Testes of Rats: A Computational and Histological Insight

**DOI:** 10.3390/jox16030095

**Published:** 2026-05-28

**Authors:** Jagjeet Singh, Annu Phogat, Reena Sheoran, Arun Hasanpuri, Vijay Kumar, Manoj Kumar Yadav, Vinay Malik

**Affiliations:** 1 Department of Zoology, Government College, Satnali, Mahendergarh 123024, Haryana, India; jagjeet.rs.zoo@mdurohtak.ac.in (J.S.); annu.rs.zoo@mdurohtak.ac.in (A.P.); 2 Department of Zoology, Maharshi Dayanand University, Rohtak 124001, Haryana, India; reena.rs.zoo@mdurohtak.ac.in (R.S.); arun.rs.zoo@mdurohtak.ac.in (A.H.); 3 Department of Biochemistry, Maharshi Dayanand University, Rohtak 124001, Haryana, India; vksiwach.biochem@mdurohtak.ac.in; 4 Department of Zoology, Rao Pahlad Singh Degree College, Balana, Mahendergarh 123029, Haryana, India; 5 Department of Genetics, Maharshi Dayanand University, Rohtak 124001, Haryana, India

**Keywords:** acetamiprid, berberine, apoptosis, testes, histology

## Abstract

Background: Acetamiprid (ACMP) exposure mediates a variety of pathological complications, including testicular toxicity. Berberine (BBR) is a plant-derived alkaloid with potential pharmacological properties. This study sought to evaluate the ameliorative effects of BBR against ACMP-induced testicular toxicity. Methods: Male Wistar rats were divided into four groups: control, BBR-treated, ACMP-exposed, and BBR+ACMP co-treated, and were administered with BBR (150 mg/kg b.wt) and ACMP (21.7 mg/kg b.wt) for 21 days. Biochemical and FTIR analyses, RT-PCR, computational analyses, and histopathological examination were conducted to assess alterations in lipid and protein profiles, as well as apoptotic and structural changes. Results: ACMP exposure was associated with oxidative injury, functional alterations (stretching of -OH, -CH_2_, -NH, C=O, C-N, -COO-, -PO_2_^−^), and compositional changes in proteins and lipids. Pre-treatment of BBR (2 h prior) was associated with attenuation of the functional and compositional alterations in proteins and lipids in co-treated rats. RT-PCR and computational analysis showed increased Bax and caspase-3 and decreased Bcl-2 mRNA expression, suggesting a potential modulation of ACMP-induced apoptosis by BBR. Histological examination showed that pre-treatment with BBR prevented ACMP-induced structural alterations, including cellular disorganization and alteration in seminiferous tubules. Conclusions: The study suggested that the BBR may exert ameliorative effects against ACMP-induced testicular toxicity by modulating lipid and protein changes and the anti-apoptotic pathway. Thus, BBR could be used as a potential ameliorative agent against oxidative stress. However, more mechanistic studies are needed for broader biological relevance and validity.

## 1. Introduction

Acetamiprid (ACMP) is a neonicotinoid insecticide broadly used in agricultural and domestic activities since the last decade [1]. At the same time, the scientific literature provided evidence of its accumulation in various environmental resources and the potential adverse effects on non-target organisms [2,3]. The main mechanisms underlying ACMP toxicity include elevated oxidative stress, apoptosis, and structural and DNA damage in mammals [4,5,6]. These degenerative changes associated with ACMP exposure translate to hepatotoxicity, neurotoxicity, nephrotoxicity, and reproductive toxicity [7,8,9,10].

Testes are considered highly vulnerable to oxidative stress due to their high levels of polyunsaturated fatty acids and functional proteins. Scientific evidence has documented that ACMP exposure is associated with oxidative stress, altered spermatogenesis, disrupted hormonal regulation and structural alterations in testicular tissue [7,11,12,13,14]. Several in vitro studies have further confirmed the pronounced toxic effects of ACMP on reproductive organs in different experimental models [3,15]. Additionally, acute and subchronic exposure to ACMP alone and in combination with other pesticides has been reported to induce apoptosis by altering Bax/Bcl-2 ratios and activating caspase-3, as well as causing DNA damage in testicular tissue [16,17,18]. Moreover, occupational exposure to ACMP has been linked with reduced sperm count, stillbirth, congenital and developmental disabilities among children born to farm workers, indicating severe reproductive toxicity of ACMP [19].

Natural antioxidants are cost-effective ameliorative agents against xenobiotic exposure-induced toxicity, with minimal side effects, and have a variety of pharmacological and clinical applications. Berberine (BBR) is an antioxidant abundant in the roots, stems, and leaves of Berberis species. Various studies have identified that the structural diversity of BBR is responsible for its therapeutic efficacy and pharmacological and pharmacokinetic properties [20,21]. Owing to these functional groups, several ameliorative potentials, such as antioxidative, anti-apoptotic, anti-inflammatory, and anti-cholinergic, are also attributed to BBR. Literature has confirmed the potential efficacy of BBR against drug abuse [22], heavy metals [23], and pesticide exposure [24] induced reproductive toxicity in various organisms. Scientific investigations have shown the ameliorative effects of BBR (50 and 100 mg/kg b.wt) against rat models for male fertility disorders induced by varicocele and gossypol exposure [25,26]. The antioxidative and anti-inflammatory properties of BBR were considered responsible for attenuating the exposure-induced reproductive toxicity.

Further, the effects of ACMP on the male reproductive system, focusing on sperm characteristics and hormonal regulations, have been documented previously. However, no study has yet applied a computational approach and examined the toxicity of ACMP with respect to the functional and molecular composition of lipids and proteins, as well as apoptotic and structural changes. The current study was conducted to explore ACMP-induced oxidative and structural damage, along with apoptotic progression, in rat testes, and to investigate the protective efficacy of BBR.

## 2. Materials and Methods

### 2.1. Reagents

Acetamiprid (C_10_H_11_ClN_4_, purity ≥ 98.0%), berberine (C_20_H_18_ClNO_4_, purity ≥ 98.0%), bovine serum albumin (BSA), 2,4-dinitrophenylhydrazine (DNPH), 5,5-dithiobis-2-nitro-benzoic acid (DTNB), trichloroacetic acid (TCA), thiobarbituric acid (TBA), guanidine hydrochloride, ethylene diamine tetraacetic acid (EDTA) and potassium bromide were purchased from Sigma-Aldrich (St. Louis, MO, USA). dNTP mix and Taq polymerase were obtained from Fisher Scientific (Mumbai, India).

### 2.2. Experimental Animals

Albino male rats (Wistar strain) weighing 150–180 g were procured from a disease-free small animal house, Lala Lajpat Rai University of Veterinary and Animal Sciences, Hisar. Rats were housed in polypropylene cages under controlled laboratory conditions, maintained at 25 ± 2 °C, 55 ± 5% humidity, and a 12 h light/dark cycle, with free access to a standard rodent pellet diet and water ad libitum. Animals were acclimatized for seven days before starting the experiment. All experimental protocols were performed in accordance with the relevant OECD, ARRIVE and Institutional Animal Ethics Committee guidelines. The utmost care was taken to ensure blinding at each stage of the experiment to avoid any selection bias.

### 2.3. Experimental Design

Experimental animals were randomly assigned to four groups containing six rats in each as follows:

- Control group: Animals were given normal saline (0.9% *w*/*v*) as a vehicle, intragastrically.
- Berberine-treated group (BBR): Animals were given 1 mL of a dose intragastrically containing 150 mg/kg b.wt BBR for 3 weeks.
- Acetamiprid-exposed group (ACMP): Animals intragastrically received 1 mL of the ACMP dose equivalent to 21.7 mg/kg b.wt (1/10th LD50 of ACMP for rats) for 3 weeks.
- Berberine–ACMP co-treated group (BBR+ACMP): Animals received BBR, and then ACMP, with a 2 h gap.

The dose of BBR was selected based on previous studies documenting its potential to mitigate tissue injury and stress-related conditions [27,28]. The dose of ACMP was selected based on an earlier study showing that 21.7 mg/kg b.wt ACMP induces hepatotoxicity in rats [29].

**Figure 1:**
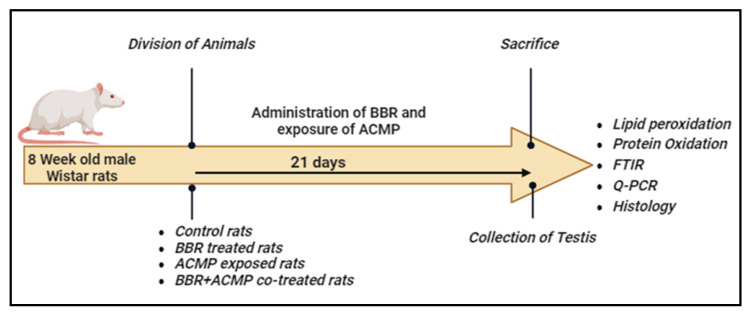
Experimental design and timeline followed during the present study.

After 24 h of the dose completion, animals were anesthetized using CO_2_ asphyxiation. For CO_2_ asphyxiation, the rats were placed in a 5 L chamber, and compressed CO_2_ was slowly released at a uniform rate (1 L/min). The rats were sacrificed via cervical dislocation. The testes were excised, rinsed with ice-cold saline, and processed for biochemical assays and Fourier transform infrared (FTIR) spectroscopy evaluation. A portion of the tissue was fixed in 10% neutral buffered formalin and processed for histopathological analysis. A part of the tissue was used for the PCR study. The schematic presentation of the experimental design and timeline followed during the present study is shown in Figure 1.

### 2.4. Preparation of Tissue Homogenate

The 500 mg of testicular tissue was homogenized in 5 mL of tissue homogenizing medium (pH 7.4) containing 0.25 M sucrose, 1 mM EDTA and 5 mM tris; using a glass Dounce homogenizer (Perfit, Gupta Scientific Industries, Ambala, India). Cellular debris was removed after centrifuging homogenate at 2100× *g* at 4 °C for 15 min, and the supernatant was again centrifuged at 13,000× *g* for 2 min. The obtained supernatant was used for biochemical assays.

### 2.5. Evaluation of Lipid Peroxidation and Protein Oxidation

Lipid peroxidation (LPO) was assayed in the obtained homogenate as described previously [30]. Oxidative damage to membrane lipids was measured spectrophotometrically by estimating malondialdehyde (MDA) content. The absorbance was recorded spectrophotometrically at 532 nm, and the results were expressed as nmol MDA/mg protein.

Oxidative damage to cellular proteins was evaluated spectrophotometrically at 370 nm by measuring the hydrazones formed from the reaction of DNPH with protein carbonyls in tissue homogenate [31]. The protein carbonyl content was expressed as nmol carbonyl/mg protein.

### 2.6. Fourier Transform Infrared Spectroscopy Analysis

For FTIR analysis, samples were prepared according to the method described previously [32]. Briefly, testis tissues were lyophilized overnight, ground, mixed with potassium bromide in a ratio of 1:100, and then pressed under a hydraulic press (1100 kg/cm^2^) to obtain pellets. FTIR spectra were recorded in the 4000–400 cm^−1^ region using an FTIR spectrophotometer (Alpha, Bruker Optics, Ettlingen, Germany). Spectra were further analyzed using OriginPro 19 (OriginLab Corporation, Northampton, MA, USA).

### 2.7. Reverse Transcription Semi-Quantitative Polymerase Chain Reaction (RT-PCR)

Total RNA was isolated using an RNA extraction kit (NP-84105, Nucleopore, New Delhi, India). The obtained RNA was checked for concentration and purity (A260/280) in a Nanodrop spectrophotometer (Denovix, Wilmington, DE, USA). Total RNA (2.5 μg) from each sample was reverse transcribed to cDNA using the RevertAid First-strand synthesis kit (Fermentas, St. Leon-Rot, Germany). Reverse transcriptase polymerase chain reaction was performed using 1 μL cDNA, DNA polymerase, and forward and reverse primers for genes of interest (Table 1) on a gradient thermal cycler (PeqStar 2X, PEQLAB, Erlangen, Germany). The reaction cycle consisted of a hot start at 95 °C for 10 min, followed by 30 cycles of amplification consisting of denaturation (94 °C, 15 s), annealing (30 s), and extension (72 °C, 30 s). The PCR products were electrophoresed on agarose gel (1.2% *w*/*v*) and photographed using the gel documentation system (XR+, Bio-Rad Laboratories, Hercules, CA, USA). Band intensities were quantified using ImageJ software (version 1.53n), with β-actin as a standard.

### 2.8. Molecular Docking

#### 2.8.1. Structure Retrieval

The 3-D crystal structures of the target proteins were obtained from the Protein Data Bank (PDB). The Bax structure was retrieved from the crystal structure of Bax (P168G) in complex with an activating antibody (PDB ID: 5W5X). The Bcl-2 protein structure was collected as the full-length Bcl complexed with the inhibitor ABT-263 (PDB ID: 4QNQ). Additionally, the structure of caspase-3 was obtained in its inactive pro-form (pro-caspase-3) with PDB ID 2J31. All structures were selected for their high resolution and biological relevance and prepared for subsequent computational analyses.

#### 2.8.2. Active Site Prediction

Active-site prediction for the selected protein structures was performed using the CASTpFold server. This automated computational tool identifies potential ligand-binding pockets based on protein surface topology and residue accessibility. The 3D structures of the Bax, Bcl-2, and caspase-3 proteins were obtained from the publicly available PDB database and submitted individually to the CASTpFold server using default parameters. The server analyzes cavity volume, pocket depth, and surface area to predict functionally relevant active/binding sites and reports the amino acid residues that constitute each predicted active pocket. The identified residues within the major binding pocket of each protein were documented for comparative analysis and further studies.

#### 2.8.3. Docking

Molecular docking was performed to predict protein-ligand interactions using the Extra Precision (XP) protocol. XP mode was selected to ensure improved pose refinement and scoring accuracy.

Ligands were prepared using the LigPrep module of Maestro, with default parameter settings, automatic ionization, generation of only 1 tautomer, and a single pose retained for each ligand. Energy minimization was performed using the OPLS3e force field. Protein structures were prepared using the Protein Preparation Wizard module, followed by an energy minimization step, which was done using the OPLS_2005 force field.

Molecular docking for Bax, Bcl-2, and caspase-3 was carried out using the same standardized workflow. Receptor grids with dimensions of 10 Å × 10 Å × 10 Å were generated by keeping the x, y, z co-ordinates (10.90, −8.93, 5.40), (4.42, 5.08, −4.70), and (−8.79, 11.84, 26.24) for Bax, Bcl-2, and caspase-3, respectively. Prior to docking, water molecules located more than 5 Å from the binding pocket were removed, whereas metal ions essential for structural integrity were preserved. Extra-precision docking was then applied to obtain refined binding poses and reliable interaction scoring.

### 2.9. Histopathological Analysis

After fixation in 10% formalin, the testes were appropriately washed under running tap water and dehydrated using an ascending series of ethyl alcohol (30%, 50%, 70%, 90%, and 100%). After dehydration, tissues were cleared in xylene, followed by xylene saturated with wax, and finally embedded in paraffin. Tissue sections of 3–5 μm were cut using a semi-automatic microtome, mounted on glass slides, and then stained with Hematoxylin and Eosin. The slides were analyzed and photographed using a digital microscope equipped with a camera (Nikon Eclipse Ci-L, Tokyo, Japan).

### 2.10. Protein Determination

The protein content was determined by the Lowry method using BSA as the standard protein [33].

### 2.11. Statistical Analysis

The results were expressed as mean ± S.D. Results were statistically analyzed using GraphPad Prism 6 software. Data were assessed for normality of distribution, and differences between groups were evaluated using Student’s *t*-test and one-way analysis of variance (ANOVA), followed by the least significant difference test, with statistical significance set at *p* ≤ 0.05 considered significant.

## 3. Results

### 3.1. Berberine Attenuated Acetamiprid-Mediated Oxidative Stress in Rat Testes

**Figure 2:**
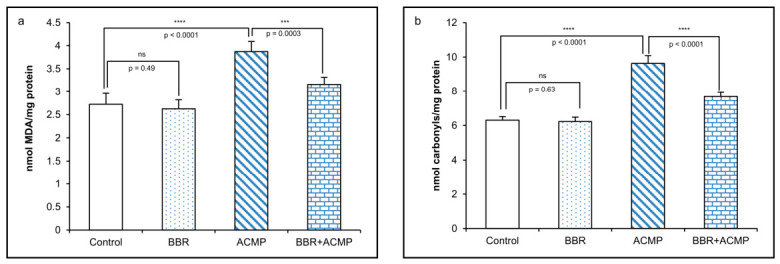
Effects of pre-treatment of berberine (BBR) and acetamiprid (ACMP) exposure on (**a**) lipid peroxidation and (**b**) protein oxidation in testes tissue of rats. Values are expressed as the mean ± S.D. (*n* = 3).

Compared with control rats, exposure to ACMP induced a significant elevation in MDA and carbonyl content in rat testes by about 42% and 53%, respectively. However, pre-treatment with BBR following ACMP exposure modulated ACMP effects on both LPO and protein oxidation, as evidenced by significant declines in MDA (27%) and carbonyl (31%) levels towards control levels. While the BBR alone treatment showed non-significant effects on both compared to the control rats (Figure 2a,b).

### 3.2. Berberine Ameliorated Acetamiprid-Induced Functional and Molecular Alterations in Lipids and Proteins in the Testes of Rats

**Figure 3:**
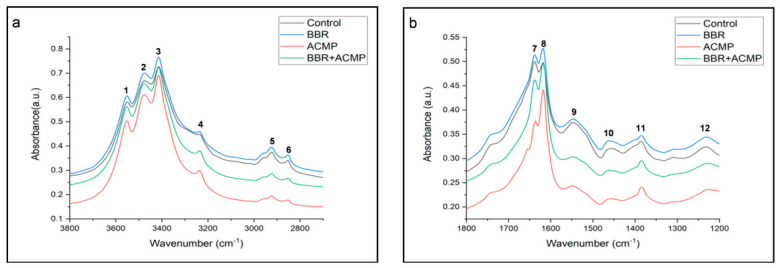
Effects of berberine (BBR) pre-treatment and acetamiprid (ACMP) exposure on functional groups of lipids and proteins in testes tissue of rats. (**a**) FTIR spectra of 3800–2700 cm^−1^ region; (**b**) FTIR spectra of 1800–1200 cm^−1^ region. The peak numbers are further detailed in Table 2.

Biological tissue consists of various macromolecules, including lipids and proteins, and possesses specific vibrational fingerprints when examined under infrared radiation. The present study investigated the effects of ACMP exposure and the ameliorative potential of a BBR supplement on the functional aspects of lipids and proteins in rat testicular tissue using FTIR spectroscopy. Figure 3a,b represent the FTIR spectrum of rat testes in different experimental groups in the 3800–2700 cm^−1^ and 1800–1200 cm^−1^ regions, respectively.

The FTIR results of the present study showed significant differences in the peak areas between control and ACMP-exposed rats, suggesting ACMP-mediated alterations in the testicular membrane. The calamitous reduction in peak areas observed at 2924 cm^−1^ (CH_2_ asymmetric stretch), 2853 cm^−1^ (CH_2_ symmetric stretch), and 1548 cm^−1^ (Amide II) following ACMP exposure indicated severe alterations in lipid flexibility and protein conformation. Further, ACMP exposure also significantly affected the area under Amide A and Amide B bands observed at 3414 cm^−1^ and 3238 cm^−1^, respectively, along with CH_2_ stretching of lipids assigned at 1459 cm^−1^, compared to the control group spectrum. Slight alterations were observed at wave numbers 3551 cm^−1^, 3475 cm^−1^ (OH stretching), and 1230 cm^−1^ (PO_2_^−^ stretching) in ACMP intoxicated groups (Table 2).

In addition, the total lipids (CH_2_ asymmetric and symmetric stretching at 2924 cm^−1^ and 2853 cm^−1^) and lipids to proteins ratio (total lipids/amide stretching at 3414 cm^−1^, 3238 cm^−1^, 1638 cm^−1^, and 1548 cm^−1^) decreased significantly in ACMP-intoxicated groups in respect to controls. Whereas a significant increase was observed in Amide I (1638 cm^−1^) to Amide II (1548 cm^−1^) and carbonyl ester (C=O stretching at 1638 cm^−1^ and 1618 cm^−1^) to total lipids ratio in ACMP exposed groups, implying the change in membrane protein conformation due to ACMP. The total lipid content in the BBR and ACMP cotreated group was similar to that of the control. The band area ratio was also significantly restored toward control levels, suggesting potential ameliorative effects of BBR against ACMP-mediated alterations in testicular membrane composition. Indeed, no significant differences were depicted in the control and BBR alone-treated groups (Table 3).

### 3.3. Berberine Protected Against Acetamiprid-Induced Apoptotic Progression in Rat Testes

**Figure 4:**
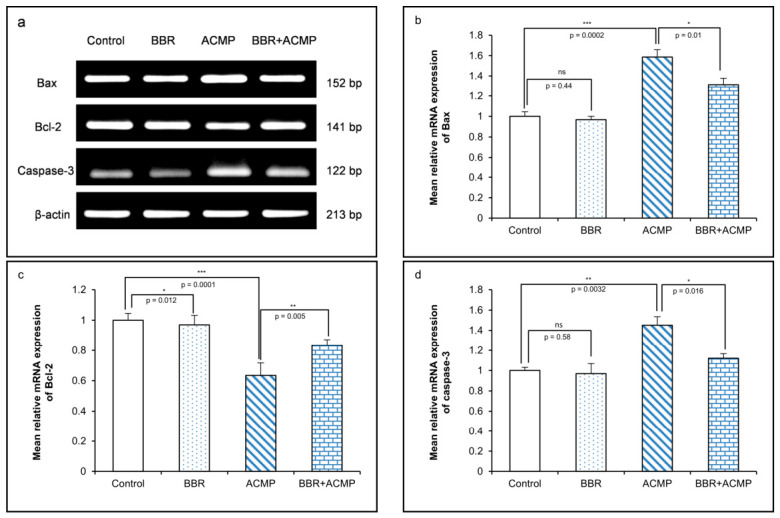
Effects of berberine (BBR) pre-treatment and acetamiprid (ACMP) exposure on mRNA expressions of apoptotic markers (**a**); Mean relative mRNA level of Bax (**b**), Bcl-2 (**c**), and caspase-3 (**d**). β-actin was used as a standard control. Values are expressed as the mean ± S.D. (*n* = 3).

Rats intoxicated with ACMP significantly upregulated the mRNA levels of Bax (53%) and caspase-3 (60%) in testes tissue. Moreover, ACMP exposure reduced Bcl-2 expression by about 33% compared with control rats, whereas 2 h prior, supplementation of BBR significantly downregulated the proapoptotic markers’ expressions (Bax by 41% and caspase-3 by 42%) and upregulated expressions of Bcl-2 (15%) compared to ACMP-exposed rats. Furthermore, BBR alone treatment didn’t alter apoptotic marker levels compared with control rats (Figure 4a–d and Figure S1).

### 3.4. Molecular Docking Results

**Figure 5:**
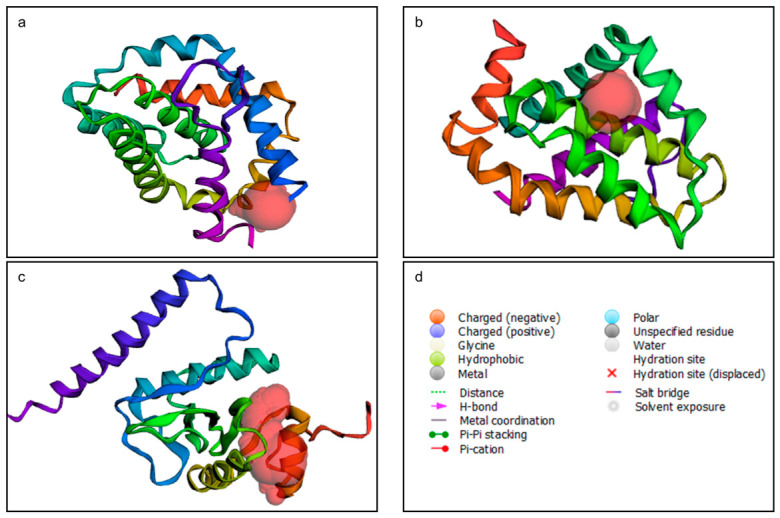
The predicted active sites of: Bax protein (**a**); Bcl-2 protein (**b**); caspase-3 protein (**c**). All the active sites are represented in red. Details of various colors and lines, along with the type of interaction they represent (**d**).

CASTpFold analysis revealed distinct and well-defined active sites in all three proteins. For Bax, the predicted active/binding site was located in Chain A and comprised residues: PRO13, GLN18, ILE19, THR22, SER55, THR56, LEU59, GLY156, GLY157, TRP158, ASP159, and LEU162. For the Bcl-2, the active/binding site was identified in Chain A and included residues: PHE97, TYR101, ALA104, PHE105, LEU108, VAL126, LEU130, ALA142, SER145, PHE146, and ALA149. Similarly, caspase-3 exhibited an extensive active site within Chain A consisting of residues PHE86, GLU89, THR90, TYR92, MET136, ASP137, TRP138, VAL139, TRP140, VAL141, GLU145, ARG146, THR148, GLY149, LEU150, PHE151, ILE167, ASP168, GLY169, PRO170, TYR171, SER172, SER174, MET175, THR177, PHE178, and ALA181. The presence of conserved aromatic, charged, and hydrophobic residues across these predicted pockets suggests their potential functional importance and supports their selection for downstream molecular docking and interaction analyses. The binding/active site of Bax, Bcl-2, and caspase-3 can be seen in the red-coloured section (Figure 5a–c, respectively). Details of distinct color codes depicted in the interaction diagrams are provided in Figure 5d.

**Figure 6:**
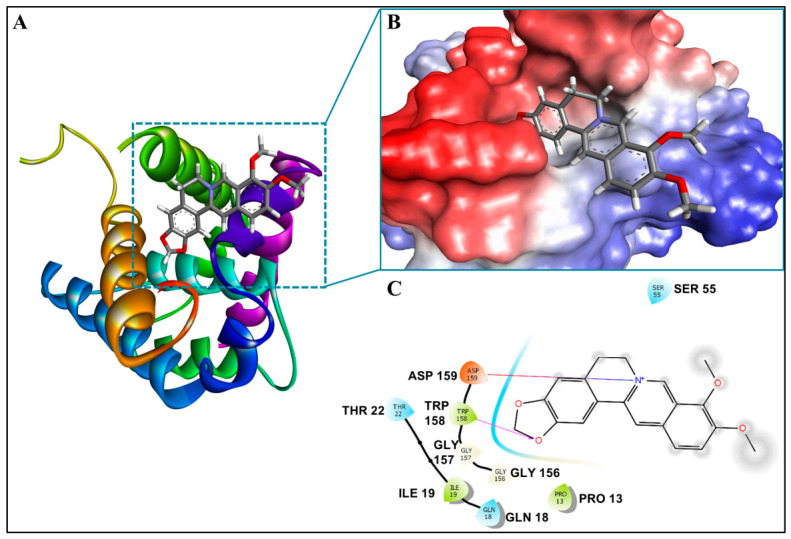
Three-dimensional interaction analysis of Bax in complex with berberine (BBR). (**A**) Cartoon representation of Bax illustrates the binding orientation of BBR within the active site. (**B**) An enlarged view of the electrostatic surface potential of Bax, emphasizing BBR encapsulated in the binding cavity. (**C**) Two-dimensional interaction diagram of Bax–BBR complex forming multiple stabilizing interactions with key amino acid residues.

**Figure 7:**
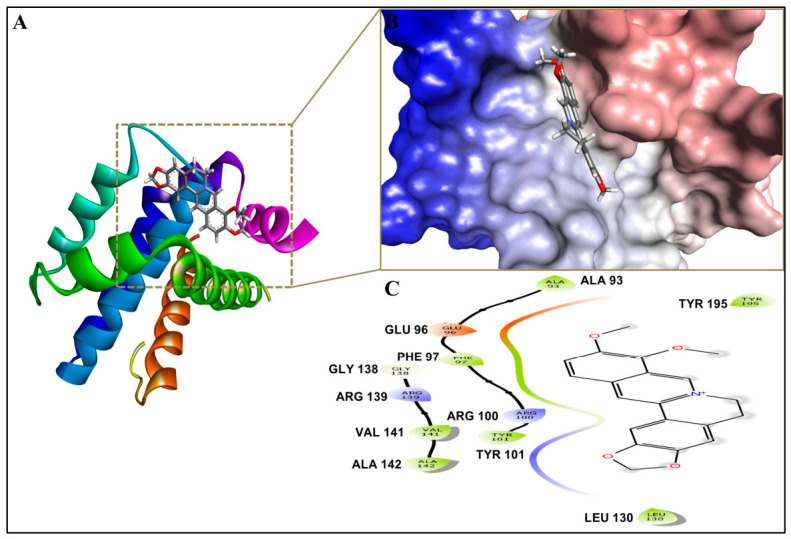
Three-dimensional interaction analysis of Bcl-2 in complex with berberine (BBR). (**A**) Cartoon representation of Bcl-2 illustrates the binding orientation of BBR within the active site. (**B**) An enlarged view of the electrostatic surface potential of Bcl-2, emphasizing BBR encapsulated in the binding cavity. (**C**) Two-dimensional interaction diagram of Bcl-2-BBR complex forming multiple stabilizing interactions with key amino acid residues.

**Figure 8:**
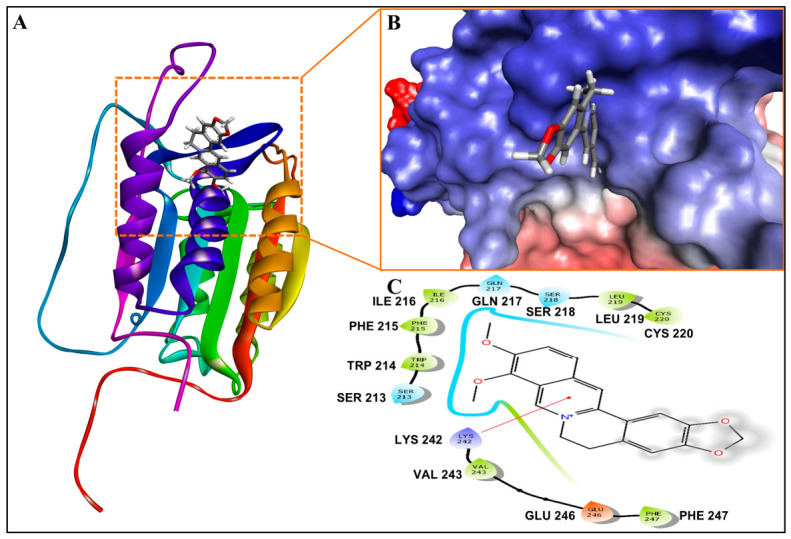
Three-dimensional interaction analysis of caspase-3 in complex with berberine (BBR). (**A**) Cartoon representation of caspase-3 illustrates the binding orientation of BBR within the active site. (**B**) An enlarged view of the electrostatic surface potential of caspase-3, emphasizing BBR encapsulated in the binding cavity. (**C**) Two-dimensional interaction diagram of caspase-3-BBR complex forming multiple stabilizing interactions with key amino acid residues.

Molecular docking was performed to evaluate the binding affinity and interaction profile of BBR with key apoptotic regulators Bax, Bcl-2, and caspase-3. For the Bax protein, BBR showed a binding affinity of −3.031 kcal/mol. The interaction profile of BBR with Bax indicates that BBR establishes a hydrogen bond with TRP158 and engages in polar interactions with THR22, GLN18, and SER55. Additionally, notable hydrophobic interactions were observed with TRP158, ILE19 and PRO13, supporting the stability of the overall ligand-protein complex (Figure 6). In the case of Bcl-2, BBR displayed a binding affinity of −3.778 kcal/mol, indicating a moderate level of interaction. The ligand predominantly exhibited hydrophobic interactions with key residues in the binding pocket, including PHE97, TYR101, LEU130, and ALA142, thereby contributing to the stability of the complex (Figure 7). Similarly, docking with caspase-3 exhibited a binding affinity of 2.868 kcal/mol, indicating a comparatively less favorable interaction. Nevertheless, BBR exhibited polar interactions with GLN217, SER218, and SER213, alongside hydrophobic contacts with ILE216, PHE215, TRP214, VAL243, PHE247, LEU219, and CYS220, suggesting partial accommodation within the caspase-3 binding pocket (Figure 8). Overall, BBR demonstrated relatively higher affinity for Bcl-2, followed by Bax, while comparatively weaker binding was observed with caspase-3, indicating selective interactions with apoptosis-related targets.

### 3.5. Berberine Prevented Acetamiprid-Induced Histopathological Changes in the Testes of Rats

**Figure 9:**
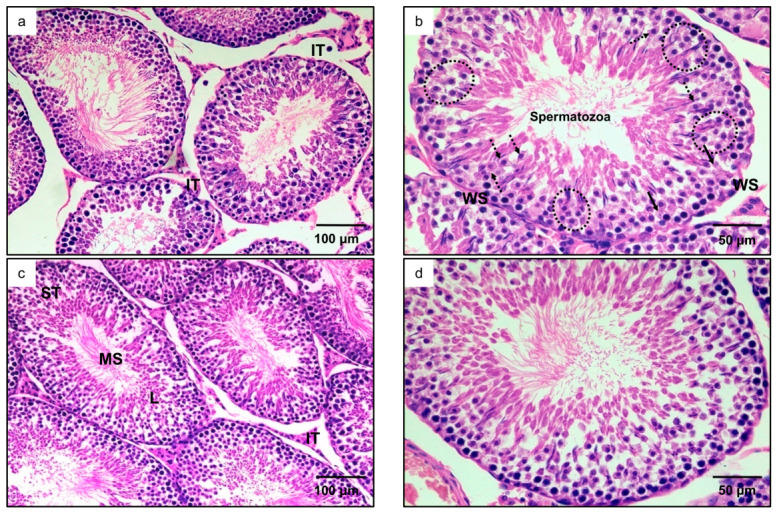
Photomicrograph of H&E-stained testes sections (200× and 400×) of control (**a**,**b**) and berberine (BBR)-treated (**c**,**d**) rats depicting standard shape of seminiferous tubules (ST), lumen (L) consisting of mature spermatozoa (MS), interstitial tissue (IT), wall of seminiferous tubule (WS), primary spermatocytes (bold arrow), nurse cells (broken arrow) and spermatids (circle).

**Figure 10:**
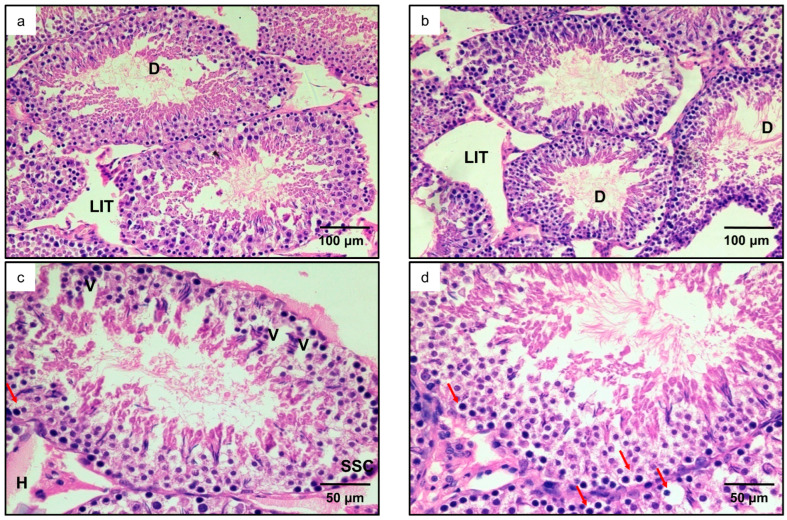
Photomicrograph of H&E-stained testes sections of acetamiprid (ACMP)-exposed rats showing decreased number of spermatozoa (D) and large intracellular spaces (LITs) at 200× (**a**,**b**); sloughing of spermatogonia cells (SSCs), haemorrhage (H), vacuolization (V), and vacuolated cytoplasm of spermatogonia (red arrow) at 400× (**c**,**d**).

**Figure 11:**
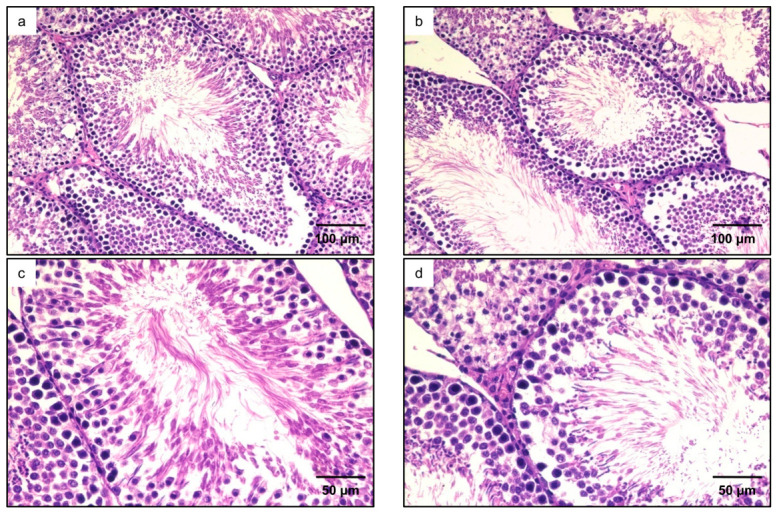
Photomicrograph of H&E-stained testes sections (200× and 400×) of berberine (BBR) and acetamiprid (ACMP) co-treated rat’s tissue sections depicting attenuation of structural changes caused by acetamiprid exposure with the regular arrangement of spermatozoa and their count in lumen (**a**–**d**).

The testicular tissue sections of control and BBR-treated rats displayed normal testicular morphology, regular thickness of the basement membrane, presence of nurse cells, and an ordered arrangement from spermatogonia to spermatids (Figure 9a–d and Figure S2). Interstitial cells appeared adjacent to seminiferous tubules. Contrary to this, ACMP administration caused severe damage, evident in the appearance of large intracellular spaces, breakage of the seminiferous tubular membrane, and a decreased number of germ cells and spermatozoa in the lumen region. Further, sloughing of spermatogenic cells, vacuolated cytoplasm of spermatogonia, and reduced numbers of spermatids were also observed in ACMP-exposed rat testes (Figure 10a–d and Figure S3). On the other hand, supplementation with BBR prevented histopathological changes in the testes of ACMP-exposed rats. Only mild vacuolization was observed in the testes of BBR and ACMP co-exposed rats (Figure 11a–d and Figure S4). The histological scoring for major structural alterations is summarised in Table 4.

## 4. Discussion

Testes are among the most essential organs, and their functioning is critical because of their role in the continuity of the species. Recent toxicological studies have confirmed the testes as the prime targeted organ during toxic exposures. Furthermore, several pesticides have been shown to cause severe alterations in testicular physiology and biochemistry, as well as detrimental effects, including apoptosis and histological changes. Thus, monitoring and evaluating the toxic effects of pesticides and exploring the therapeutic potential of some natural supplements have been much sought after. The present study investigated the ameliorative and preventive potential of BBR against ACMP-induced toxicity in rat testes.

Acetamiprid is known to cause toxicity in different organs of mammals. Occupational or environmental exposure to ACMP has been reported to affect sperm production, reduce sperm count, and decrease male fertility [13,34]. The mechanism of ACMP-induced testicular toxicity is supposed to be associated with oxidative stress and inherent biochemical changes to lipids and proteins.

The results of the present study corroborate earlier studies demonstrating that ACMP exposure induces oxidative injury [13,14]. Lipid peroxidation is considered a triggering point for ACMP toxicity in living issues. Testes are highly prone to lipid peroxidation due to high levels of polyunsaturated lipids [18]. Furthermore, lipid peroxidation produces free radicals, leading to oxidative stress and the attack on other macromolecules. The results of the present study showed an elevation in lipid peroxidation, suggesting the involvement of oxidative stress in ACMP-mediated testicular toxicity. In contrast, supplementation with BBR significantly reduced lipid peroxidation, highlighting its antioxidative potential. BBR has been reported to quench 1,1-diphenyl-1-picrylhydrazyl radical, which is mainly responsible for causing oxidative injury in lipids in rat liver [35]. This can be attributed to BBR’s antioxidant activity, which mitigates testicular toxicity. Our results are consistent with earlier findings [36], which reported that BBR protects against diclofenac-induced oxidative injury in rat testicular tissue through its free radical-scavenging activity. Further, BBR has also been shown to inhibit lipid peroxidation in mercury chloride-mediated testicular impairments in mice via its antioxidative activity [23]. Protein oxidation is another indicator of oxidative stress within tissues [37]. An increase in protein carbonyls indicates either oxidation of amino acids or cleavage of the protein backbone. The present study showed a marked increase in protein oxidation in testicular tissue following ACMP exposure, which agrees with previous studies reporting protein oxidation in rat liver [38] and brain [10]. However, administration of BBR ameliorated elevated protein oxidation in rat testicular tissue. The results are consistent with previous studies reporting that BBR and other natural antioxidants can attenuate protein oxidation by quenching free radicals and increasing endogenous antioxidants [6,39,40].

FTIR spectroscopy can reflect the molecular and structural composition of biomolecules, especially lipids and proteins. The shift in peak positions and areas provides valuable information about the structure, conformation, and function of biological molecules [41]. So, FTIR was used to elucidate conformational changes in tissues, as lipid and protein content are crucial for determining tissue morphology and physiology [32]. The amide I band (C=O stretching in the amide group) is highly sensitive to hydrogen bonding, and the Amide II band (N-H bending and C-N stretching) is an essential marker for protein structure [42]. The reduced peak areas of the Amide I and Amide II bands reflected conformational disorders in the protein backbone and decreased protein synthesis following ACMP exposure. It could be elucidated that ACMP-generated free radicals might have caused protein degradation, possibly by increasing the accessibility of protein bonds.

Additionally, changes in the asymmetric and symmetric CH_2_ stretching modes of lipids provide information about the conformation and flexibility of the lipid acyl chain [43,44]. Acetamiprid exposure significantly decreased their band areas, suggesting alterations in lipid dynamics in the ACMP-exposed testes tissue. Earlier, lipid peroxidation was reported to be a prime cause of altering the fluidity and dynamics of various biomembranes, findings corroborated by results from this study [45,46]. Further, ACMP-mediated lipid peroxidation was also evident in the reduced peak area of the CH_2_ stretching band.

The area obtained from the vibration of a specific molecule is directly proportional to the concentration of specific functional groups [47]. So, to analyze compositional and structural alterations in membrane lipids and proteins, the area ratios of specific bands were calculated. FTIR analysis revealed a decrease in the lipid-to-protein ratio in ACMP-intoxicated groups, indicating membrane dysfunction. The drastic decrease in total lipids and the increase in the carbonyl ester-to-lipid suggested that ACMP might have either reduced membrane thickness or induced severe lipid peroxidation, thereby altering the composition and distribution of lipids in the testicular membrane. In addition, a significant increase in the band area ratio of Amide I to Amide II depicted alterations in the secondary structure of proteins. This increase might be due to ACMP-mediated disruption of membrane proteins, altering their conformation. The observed results are consistent with the biochemical findings of our study. However, BBR supplementation before ACMP exposure restored the area under different spectral bands. The results suggest that the free radical scavenging potential and membrane-stabilizing efficacy of BBR might be responsible for its protective actions on lipid and protein molecular structures.

Biochemical changes involving functional and structural alterations in lipids and protein chains indicate the progression of apoptosis. The present study reported that exposure to ACMP increased mRNA expressions of Bax and caspase-3 and downregulated mRNA expression of Bcl-2 in the testes of rats. However, the RT-PCR results of the present study suggest an association with apoptotic modulations. Bax is a proapoptotic protein that initiates apoptotic progression, while caspase-3 marks the completion of apoptosis inside the tissue. Earlier studies showed that caspase-3 levels were elevated as an apoptosis marker in rat brain, kidney and testicular tissues [16,17,48,49]. Additionally, an in vitro study also reported increased Bax expression and decreased Bcl-2 expression in trophoblast cells [50], which concurs with the present results. On the other hand, administration of BBR significantly diminished the elevated mRNA expression of Bax and caspase-3 and increased Bcl-2 levels in the testes of ACMP-exposed rats. These results are consistent with an earlier study demonstrating the restoration of the Bax/Bcl-2 ratio and caspase-3 levels in rat tissues following mercury exposure [51,52].

Furthermore, a computational molecular docking analysis was performed to assess the binding affinity of BBR to multiple protein targets. This approach enables the prediction of non-covalent interactions and the identification of specific binding sites on the proteins. The docking analyses revealed distinct interaction profiles for BBR with apoptosis-related proteins. Notably, BBR displayed the highest binding affinity for Bcl-2, followed by Bax, while its affinity for caspase-3 was comparatively lower. The Bcl-2-BBR complex is characterized by a predominance of hydrophobic interactions, which likely stabilize ligand binding. In contrast, the Bax–BBR interaction involves both hydrogen bonding and a combination of polar and hydrophobic contacts, suggesting a moderately stable interaction. Despite the reduced binding affinity observed with caspase-3, the interaction suggests some level of accommodation of BBR within the predicted binding pocket. It is important to note that these findings are exploratory in nature and should not be viewed as definitive mechanistic insights. Overall, the data indicate a preferential interaction of BBR with Bcl-2 and Bax, yet the moderate binding affinities and the inherent limitations of docking methodologies necessitate a cautious interpretation of these results as preliminary.

Histopathological examination reflects the internal state of order of tissues and cells comprising a tissue. In the present study, histopathological examination revealed that ACMP exposure reduced spermatozoa, increased the appearance of large intercellular spaces and vacuolation, disrupted tubular membranes and reduced spermatogonia, findings are in line with previous studies [12,13]. The observed structural alterations correlate with this study’s biochemical, FTIR, and RT-PCR findings. Meanwhile, the administration of BBR in BBR+ACMP co-treated rats attenuated tubular membrane injury, prevented sperm loss, and restored the number of spermatogonia. The findings indicate BBR’s antioxidative and anti-apoptotic effects. The results of the present study agree with some previous studies documenting the ameliorative potential of BBR against pesticide- and drug-mediated structural alterations in testicular tissues [24,53]. Moreover, a study also demonstrated the dose-dependent protective efficacy of BBR against testicular histopathological alterations induced by ischemic/reperfusion injury [54].

## 5. Conclusions

Collectively, our results showed that ACMP exposure induces severe oxidative stress in rat testes, resulting in lipid peroxidation, protein oxidation, apoptosis, and marked histological alterations. The apoptotic effects and structural alterations are likely mediated through oxidative stress-associated dysregulation of the Bax/Bcl-2 signaling axis. Pre-treatment with BBR alleviated the toxicity of ACMP exposure in the testes. BBR exhibited potential ameliorative properties against LPO, protein oxidation and structural changes by preventing functional alterations in the lipid or protein backbone and modulating the apoptotic cascade.

The findings suggest that BBR exerts potent antioxidant and anti-apoptotic effects against pesticide-induced reproductive toxicity and may serve as a promising therapeutic molecule for mitigating testicular oxidative injury. This study also has clinical implications, mainly because numerous people are exposed to pesticides, primarily during agricultural activities. However, efficient therapies remain an unmet need, and there is an urgent need for more mechanistic research to yield more valid, quantified results that delineate the molecular mechanisms underlying BBR and other bioactive therapeutic molecules.

## Figures and Tables

**Table 1:** Details of the primer used for RT-PCR analysis in this study.

Primer	Accession	Sequence (5′–3′)	Size (bp)
Caspase-3	NM_012922.2	F-GACAACAACGAAACCTCC	122
		R-AGGGTAATCCTTTTGTAACTG	
Bax	NM_017059.2	F-GGCTGGACACTGGACTTC	152
		R-CAGATGGTGAGTGAGGCA	
Bcl-2	NM_016993.2	F-GTGGACAACATCGCTCTG	141
		R-AGACAGCCAGGAGAAATCA	
β-actin	V01217.1	F-TTGCCCTAGACTTCGAGCAA	213
		R-AGACTTACAGTGTGGCCTCC	

**Table 2:** Description of frequency assignment and effects of berberine pre-treatment and acetamiprid exposure on peak area value in testes tissue of different experimental groups.

S. No	Peak	Peak Area				Associated Functional Group
		Control	BBR	ACMP	BBR+ACMP	
1	3551	27.26 ± 0.28	27.74 ± 0.55	31.58 ± 0.20 ***	29.72 ± 0.16 ^###^	O-H stretching
2	3475	29.57 ± 0.62	29.88 ± 0.81	34.41 ± 0.33 **	31.14 ± 0.20 ^###^	O-H stretching
3	3414	57.17 ± 0.77	57.11 ± 0.70	56.35 ± 0.25	55.36 ± 1.01	Amide A: N-H stretching
4	3238	22.01 ± 0.42	22.74 ± 0.41	15.37 ± 0.30 ***	19.05 ± 0.22 ^###^	Amide B: N-H stretching
5	2924	8.76 ± 0.39	9.24 ± 0.50	3.86 ± 0.17 ***	4.76 ± 0.31 ^#^	CH_2_ asymmetric stretching of lipids
6	2853	4.75 ± 0.27	5.23 ± 0.44	1.86 ± 0.27 ***	3.05 ± 0.19 ^##^	CH_2_ symmetric stretching of lipids
7	1638	15.19 ± 0.34	14.72 ± 0.28	9.80 ± 0.13 ***	11.29 ± 0.23 ^##^	Amide I: C=O stretching
8	1618	7.32 ± 0.14	7.31 ± 0.06	7.26 ± 0.13	7.35 ± 0.14	C=O stretching of proteins
9	1548	6.81 ± 0.32	6.34 ± 0.30	2.98 ± 0.13 ***	4.58 ± 0.22 ^##^	Amide II: N-H bend and C-N stretch
10	1459	2.14 ± 0.10	2.04 ± 0.15	0.87 ± 0.07 ***	1.09 ± 0.16	CH_2_ stretching bend of lipids
11	1385	3.83 ± 0.25	3.63 ± 0.29	2.26 ± 0.10 **	2.98 ± 0.19 ^#^	COO- a symmetric stretch of fatty acid
12	1230	4.50 ± 0.22	4.36 ± 0.08	3.26 ± 0.16 **	4.00 ± 0.16 ^##^	PO_2_^−^ asymmetric stretching: Phosphoric acid and nucleic acids

Values are represented as mean ± S.D. (*n* = 3). ** *p* ≤ 0.01, and *** *p* ≤ 0.001 are statistically different from the control group; ^#^ *p* ≤ 0.05, ^##^ *p* ≤ 0.01, and ^###^ *p* ≤ 0.001 are statistically different from ACMP-exposed group.

**Table 3:** Description of various functional attributes of lipids and proteins in different experimental groups.

Functional Group	Control	BBR	ACMP	BBR+ACMP
Total Lipids	13.51 ± 0.35	14.47 ± 0.93	05.72 ± 0.41 ***	07.82 ± 0.47 ^##^
Lipid/Proteins	0.134 ± 0.004	0.143 ± 0.009	0.068 ± 0.005 ***	0.087 ± 0.004 ^##^
Carbonyl ester/Total Lipids	1.67 ± 0.03	1.53 ± 0.12	2.99 ± 0.25 ***	2.39 ± 0.15 ^#^
Amide I/Amide II	2.23 ± 0.06	2.32 ± 0.07	3.29 ± 0.14 ***	2.47 ± 0.08 ^##^

Values are represented as mean ± S.D. (*n* = 3). *** *p* ≤ 0.001 is statistically different from the control group; ^#^ *p* ≤ 0.05 and ^##^ *p* ≤ 0.01 are statistically different from ACMP-exposed group.

**Table 4:** Description of major histological alterations and Johnson-like scoring.

Damage to	Control	BBR	ACMP	BBR+ACMP
Spermatogonia	-	-	++	-
Primary Spermatocytes	-	-	++	-
Spermatids	-	-	+++	+
Spermatozoa	-	-	+++	++
Johnson-like Scoring	9.4 ± 0.49	9.6 ± 0.49	7.6 ± 0.8 *	9 ± 0.63 ^#^

(-) No change; (+) mild alteration; (++) moderate alterations; and (+++) severe alterations * *p* ≤ 0.05 is statistically different from the control group; ^#^ *p* ≤ 0.05 is statistically different from ACMP-exposed group.

## Data Availability

The original contributions presented in this study are included in the article/Supplementary Materials. Further inquiries can be directed to the corresponding authors.

## Supplementary Materials

The following supporting information can be downloaded at: https://www.mdpi.com/article/10.3390/jox16030095/s1, Figure S1: Original gel images depicted in Figure 4a; Figure S2: Unlabelled images of histology depicted in Figure 9; Figure S3: Unlabelled images of histology depicted in Figure 10; Figure S4: Unlabelled images of histology depicted in Figure 11.

## Abbreviations

The following abbreviations are used in this manuscript:

BBR	Berberine
ACMP	Acetamiprid
FTIR	Fourier transform infrared
b.wt	Body weight

## References

[1] Phogat A., Singh J., Kumar V., Malik V. (2022). Toxicity of the Acetamiprid Insecticide for Mammals: A Review. Environ. Chem. Lett..

[2] Craddock H.A., Huang D., Turner P.C., Quirós-Alcalá L., Payne-Sturges D.C. (2019). Trends in Neonicotinoid Pesticide Residues in Food and Water in the United States, 1999–2015. Environ. Health.

[3] Terayama H., Qu N., Endo H., Ito M., Tsukamoto H., Umemoto K., Kawakami S., Fujino Y., Tatemichi M., Sakabe K. (2018). Effect of Acetamiprid on the Immature Murine Testes. Int. J. Environ. Health Res..

[4] Annabi E., Ben Salem I., Abid-Essefi S. (2019). Acetamiprid, a Neonicotinoid Insecticide, Induced Cytotoxicity and Genotoxicity in PC12 Cells. Toxicol. Mech. Methods.

[5] Kara M., OztaŞ E., Özhan G. (2020). Acetamiprid-Induced Cyto- and Genotoxicity in the AR42J Pancreatic Cell Line. Turk. J. Pharm. Sci..

[6] Phogat A., Singh J., Kumar V., Malik V. (2023). Berberine Mitigates Acetamiprid-Induced Hepatotoxicity and Inflammation via Regulating Endogenous Antioxidants and NF-κB/TNF-α Signaling in Rats. Environ. Sci. Pollut. Res..

[7] Zhang J., Wang Y., Xiang H., Li M.-X., Li W.-H., Wang X., Zhang J. (2011). Oxidative Stress: Role in Acetamiprid-Induced Impairment of the Male Mice Reproductive System. Agric. Sci. China.

[8] Rasgele P.G., Oktay M., Kekecoglu M., Muranli F.D.G. (2015). The Histopathological Investigation of Liver in Experimental Animals after Short-Term Exposures to Pesticides. Bulg. J. Agric. Sci..

[9] Devan R.S., Mishra A., Prabu P.C., Mandal T.K., Panchapakesan S. (2015). Sub-Chronic Oral Toxicity of Acetamiprid in Wistar Rats. Toxicol. Environ. Chem..

[10] Phogat A., Singh J., Malik V., Kumar V. (2023). Neuroprotective Potential of Berberine Against Acetamiprid Induced Toxicity in Rats: Implication of Oxidative Stress, Mitochondrial Alterations, and Structural Changes in Brain Regions. J. Biochem. Mol. Toxicol..

[11] Chawseen M.A. (2011). Effects of Acetamiprid and Glyphosate Pesticides on Testis and Serum Testosterone Level in Male Mice. J. Duhok Univ..

[12] Zayman E., Gül M., Erdemli M.E., Gül S., Bağ H.G., Taşlıdere E. (2022). Biochemical and Histopathological Investigation of the Protective Effects of Melatonin and Vitamin E Against the Damage Caused by Acetamiprid in Balb-c Mouse Testicles at Light and Electron Microscopic Level. Environ. Sci. Pollut. Res. Int..

[13] Arıcan E.Y., Gokçeoglu Kayalı D., Ulus Karaca B., Boran T., Oturk N., Okyar A., Ercan F., Ozhan G. (2020). Reproductive Effects of Subchronic Exposure to Acetamiprid in Male Rats. Sci. Rep..

[14] El-Hak H.N.G., Al-Eisa R.A., Ryad L., Halawa E., El-Shenawy N.S. (2022). Mechanisms and Histopathological Impacts of Acetamiprid and Azoxystrobin in Male Rats. Environ. Sci. Pollut. Res..

[15] Kenfack A., Guiekep N.A.J., Ngoula F., Vemo B.N., Bouli E.P., Pamoal E.T. (2018). Reproductive Toxicity of Acetamiprid in Male Guinea Pig (*Cavia porcellus*). J. Anim. Sci. Vet. Med..

[16] El-Demerdash F.M., Fayed M.Y., Tousson E.M., El-Sayed R.A. (2025). Omega-3 Fatty Acids Modulate Acetamiprid and Emamectin Benzoate-Induced Testicular Toxicity in Rats by Modulating Nrf2/NFkB Pathway and Apoptotic Signaling. Sci. Rep..

[17] Demir S., Alemdar N.T., Yulug E., Kulaber A., Demir E.A., Erdogan N.S., Mentese A., Aliyazicioglu Y. (2026). Chrysin Mitigates Acetamiprid-Induced Testicular Injury in Mice via Suppression of Endoplasmic Reticulum Stress and Inflammation. Environ. Toxicol..

[18] Fouda H.M., Yosef T.A., Abd El-Rheem S.M., Almadaly E.A., Hegazy H.M. (2026). Protective Effect of Chlorella Vulgaris on Sub-Chronic Hepatic and Testicular Toxicity Induced by Acetamiprid in Rats. Front. Vet. Sci..

[19] Neghab M., Momenbella-Fard M., Naziaghdam R., Salahshour N., Kazemi M., Alipour H. (2014). The Effects of Exposure to Pesticides on the Fecundity Status of Farm Workers Resident in a Rural Region of Fars Province, Southern Iran. Asian Pac. J. Trop. Biomed..

[20] Gaba S., Saini A., Singh G., Monga V. (2021). An Insight into the Medicinal Attributes of Berberine Derivatives: A Review. Bioorg. Med. Chem..

[21] Singh S., Pathak N., Fatima E., Negi A.S. (2021). Plant Isoquinoline Alkaloids: Advances in the Chemistry and Biology of Berberine. Eur. J. Med. Chem..

[22] Altunas H., Ozdemir M., Harmanci N., Yigitaslan S., Sahinturk V. (2023). The Effect of Berberine on the Prevention and/or Treatment on Cyclophosphamide-Induced Testicular Damage in Rats. Osman. Tıp Derg..

[23] Albasher G., Alkahtani S., Alarifi S. (2020). Berberine Mitigates Oxidative Damage Associated with Testicular Impairment Following Mercury Chloride Intoxication. J. Food Biochem..

[24] Murad H.M., Abdulameer S.A., Aljuboory D.S.A., Neamah D.A., Hamza A. (2020). Defensive Effects of Breberine Against Cypermethrin Induced Male Reproductive System Toxicity in Rabbits. Syst. Rev. Pharm..

[25] Saleh S.R., Attia R., Ghareeb D.A. (2018). The Ameliorating Effect of Berberine-Rich Fraction Against Gossypol-Induced Testicular Inflammation and Oxidative Stress. Oxid. Med. Cell. Longev..

[26] Rashtbari H., Razi M., Hassani-Bafrani H., Najaran H. (2018). Berberine Reinforces Sertoli Cells Niche and Accelerates Spermatogonial Stem Cells Renewal in Experimentally-Induced Varicocele Condition in Rats. Phytomedicine.

[27] Li D., Zheng J., Hu Y., Hou H., Hao S., Liu N., Wang Y. (2017). Amelioration of Intestinal Barrier Dysfunction by Berberine in the Treatment of Nonalcoholic Fatty Liver Disease in Rats. Pharmacogn. Mag..

[28] Zhao L., Cang Z., Sun H., Nie X., Wang N., Lu Y. (2017). Berberine Improves Glucogenesis and Lipid Metabolism in Nonalcoholic Fatty Liver Disease. BMC Endocr. Disord..

[29] Chakroun S., Ezzi L., Grissa I., Kerkeni E., Neffati F., Bhouri R., Sallem A., Najjar M.F., Hassine M., Mehdi M. (2016). Hematological, Biochemical, and Toxicopathic Effects of Subchronic Acetamiprid Toxicity in Wistar Rats. Environ. Sci. Pollut. Res..

[30] Prakash C., Kamboj V.K., Ahlawat P., Kumar V. (2015). Structural and Molecular Alterations in Arsenic-Induced Hepatic Oxidative Stress in Rats: A FTIR Study. Toxicol. Environ. Chem..

[31] Singh J., Phogat A., Prakash C., Chhikara S.K., Singh S., Malik V., Kumar V. (2021). N-Acetylcysteine Reverses Monocrotophos Exposure-Induced Hepatic Oxidative Damage via Mitigating Apoptosis, Inflammation and Structural Changes in Rats. Antioxidants.

[32] Akkas S.B., Severcan M., Yilmaz O., Severcan F. (2007). Effects of Lipoic Acid Supplementation on Rat Brain Tissue: An FTIR Spectroscopic and Neural Network Study. Food Chem..

[33] Lowry O.H., Rosebrough N.J., Farr A.L., Randall R.J. (1951). Protein Measurement with the Folin Phenol Reagent. J. Biol. Chem..

[34] Kong D., Zhang J., Hou X., Zhang S., Tan J., Chen Y., Yang W., Zeng J., Han Y., Liu X. (2017). Acetamiprid Inhibits Testosterone Synthesis by Affecting the Mitochondrial Function and Cytoplasmic Adenosine Triphosphate Production in Rat Leydig Cells. Biol. Reprod..

[35] Hwang J.-M., Wang C.-J., Chou F.-P., Tseng T.-H., Hsieh Y.-S., Lin W.-L., Chu C.-Y. (2002). Inhibitory Effect of Berberine on Tert-Butyl Hydroperoxide-Induced Oxidative Damage in Rat Liver. Arch. Toxicol..

[36] Waly H., Abd-Elkareem M., Raheem S.A., Abou Khalil N.S. (2022). Berberine Protects Against Diclofenac Sodium-Induced Testicular Impairment in Mice by Its Anti-Oxidant and Anti-Apoptotic Activities. Iran. J. Basic Med. Sci..

[37] Deavall D.G., Martin E.A., Horner J.M., Roberts R. (2012). Drug-Induced Oxidative Stress and Toxicity. J. Toxicol..

[38] Phogat A., Singh J., Sheoran R., Hasanpuri A., Malik V. (2024). Acetamiprid Exposure Causes Molecular and Structural Changes in the Liver and Kidney Tissues of Rats. Chem. Biol. Lett..

[39] El-Bialy B.E.S., Abd Eldaim M.A., Hassan A., Abdel-Daim M.M. (2020). Ginseng Aqueous Extract Ameliorates Lambda-Cyhalothrin-Acetamiprid Insecticide Mixture for Hepatorenal Toxicity in Rats: Role of Oxidative Stress-Mediated Proinflammatory and Proapoptotic Protein Expressions. Environ. Toxicol..

[40] Malik V., Singh J., Kumar A., Kumar V. (2021). Protective Effect of Coenzyme Q10 Nanoparticles Against Monocrotophos Induced Oxidative Stress in Kidney Tissues of Rats. Biologia.

[41] Severcan F., Haris P.I. (2012). Vibrational Spectroscopy in Diagnosis and Screening.

[42] Haris P.I., Severcan F. (1999). FTIR Spectroscopic Characterization of Protein Structure in Aqueous and Non-Aqueous Media. J. Mol. Catal. B Enzym..

[43] Liu K.-Z., Bose R., Mantsch H.H. (2002). Infrared Spectroscopic Study of Diabetic Platelets. Vib. Spectrosc..

[44] Toyran N., Zorlu F., Dönmez G., Öğe K., Severcan F. (2004). Chronic Hypoperfusion Alters the Content and Structure of Proteins and Lipids of Rat Brain Homogenates: A Fourier Transform Infrared Spectroscopy Study. Eur. Biophys. J..

[45] García J.J., Reiter R.J., Guerrero J.M., Escames G., Byung P.Y., Oh C.S., Muñoz-Hoyos A. (1997). Melatonin Prevents Changes in Microsomal Membrane Fluidity during Induced Lipid Peroxidation. FEBS Lett..

[46] Karbownik M., Reiter R.J. (2000). Antioxidative Effects of Melatonin in Protection Against Cellular Damage Caused by Ionizing Radiation. Proc. Soc. Exp. Biol. Med. Minireviews.

[47] Cakmak G., Zorlu F., Severcan M., Severcan F. (2011). Screening of Protective Effect of Amifostine on Radiation-Induced Structural and Functional Variations in Rat Liver Microsomal Membranes by FT-IR Spectroscopy. Anal. Chem..

[48] Gasmi S., Chafaa S., Lakroun Z., Rouabhi R., Touahria C., Kebieche M., Soulimani R. (2019). Neuronal Apoptosis and Imbalance of Neurotransmitters Induced by Acetamiprid in Rats. Toxicol. Environ. Health Sci..

[49] Erdemli M.E., Zayman E., Erdemli Z., Gul M., Gul S., Gozukara Bag H. (2020). Protective Effects of Melatonin and Vitamin E in Acetamiprid-Induced Nephrotoxicity. Environ. Sci. Pollut. Res..

[50] Gomez S.D., Bustos P.S., Sánchez V.G., Ortega M.G., Guiñazú N. (2020). Trophoblast Toxicity of the Neonicotinoid Insecticide Acetamiprid and an Acetamiprid-Based Formulation. Toxicology.

[51] Abdel Moneim A.E. (2015). The Neuroprotective Effect of Berberine in Mercury-Induced Neurotoxicity in Rats. Metab. Brain Dis..

[52] Othman M.S., Safwat G., Aboulkhair M., Abdel Moneim A.E. (2014). The Potential Effect of Berberine in Mercury-Induced Hepatorenal Toxicity in Albino Rats. Food Chem. Toxicol..

[53] Sharma R., Goyal N., Singla M., Sharma V.L. (2019). Berberis Aristata Ameliorates Testicular Toxicity Induced by Combination of First-Line Tuberculosis Drugs (Rifampicin + Isoniazid + Pyrazinamide) in Normal Wistar Rats. J. Diet. Suppl..

[54] Moradi-Ozarlou M., Ashrafizadeh M., Javanmardi S. (2021). The Ameliorative Impacts of Berberine on Testicular Ischemia/Reperfusion Injury in Rats: An Experimental Study. Iran. J. Vet. Surg..

