# Supplementary material for: Ameliorative Effects of Berberine Against Acetamiprid-Induced Toxicity in the Testes of Rats: A Computational and Histological Insight
Source: J Xenobiot. 2026 May 28;16(3):95. doi: 10.3390/jox16030095 (PMC13302651; doi:10.3390/jox16030095)
Supplement: Supplementary file 1 [file jox-16-00095-s001.zip › jox-4270807-supplementary.pdf]

Supplementary Materials: Ameliorative Effects of Berberine Against Acetamiprid-Induced Toxicity in the Testes of Rats:

A Computational and Histological Insight

Jagjeet Singh, Annu Phogat, Reena Sheoran, Arun Hasanpuri, Vijay Kumar, Manoj Kumar Yadav , and Vinay Malik

Figure S1: Original gel images depicted in Figure 4a.

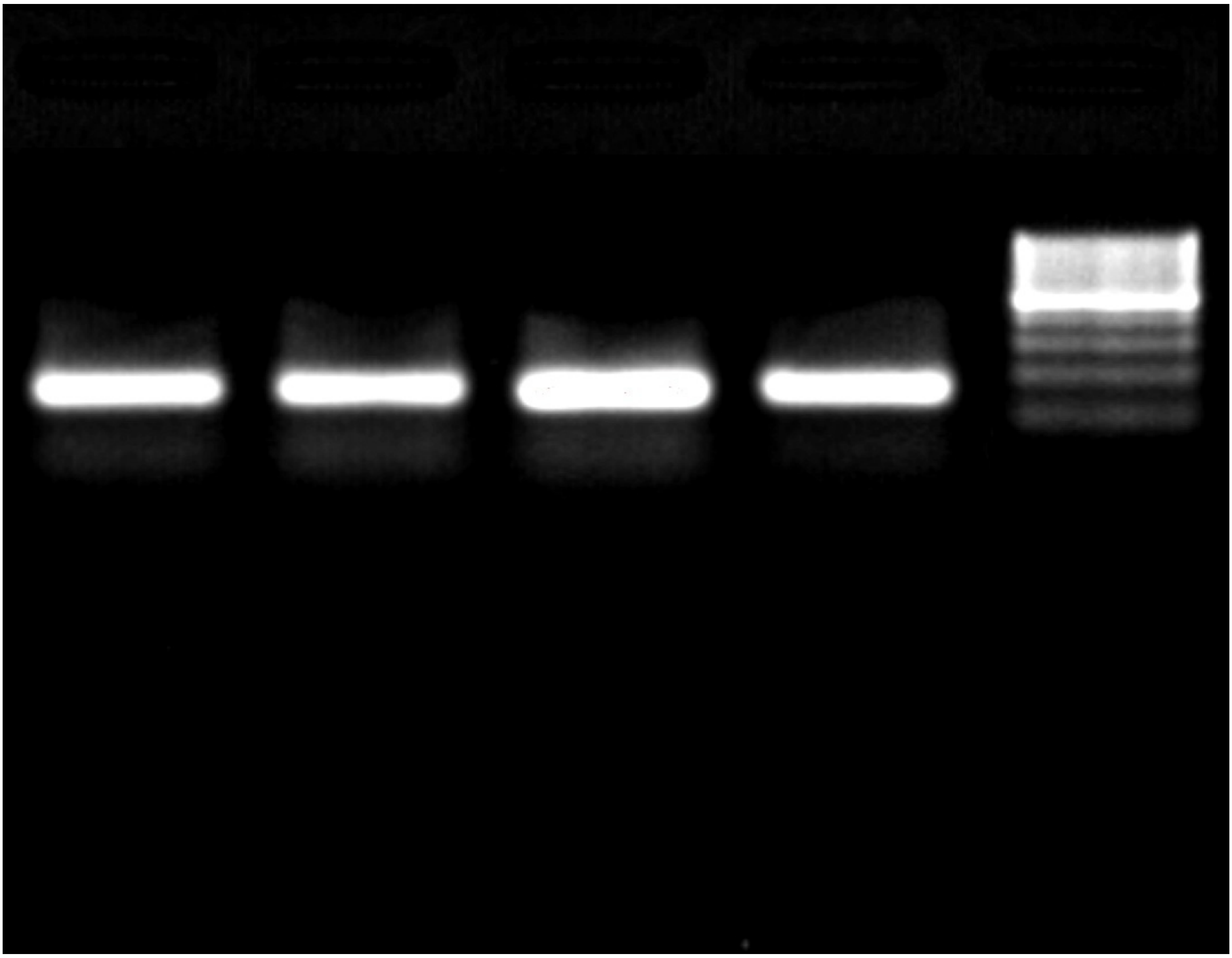

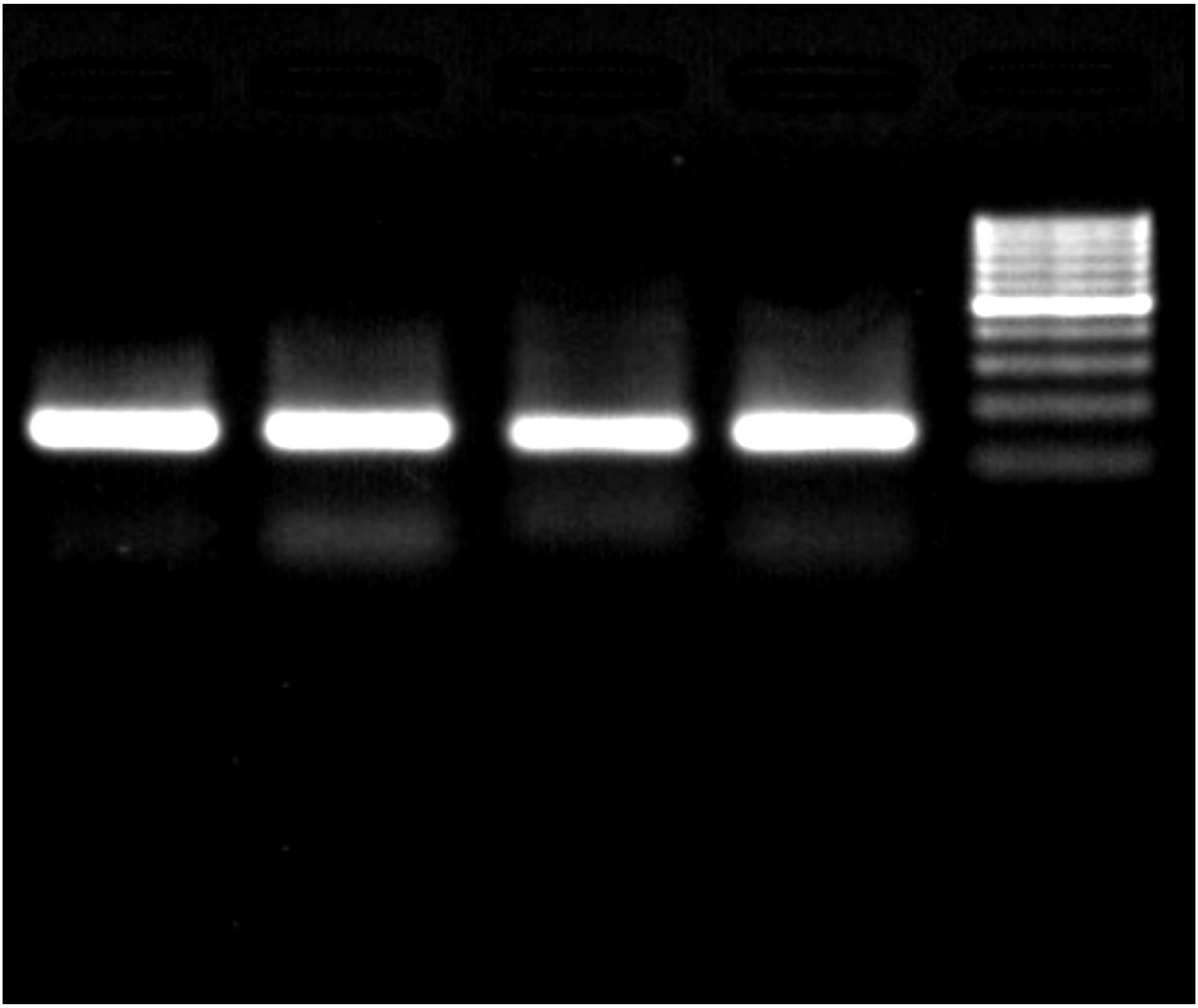

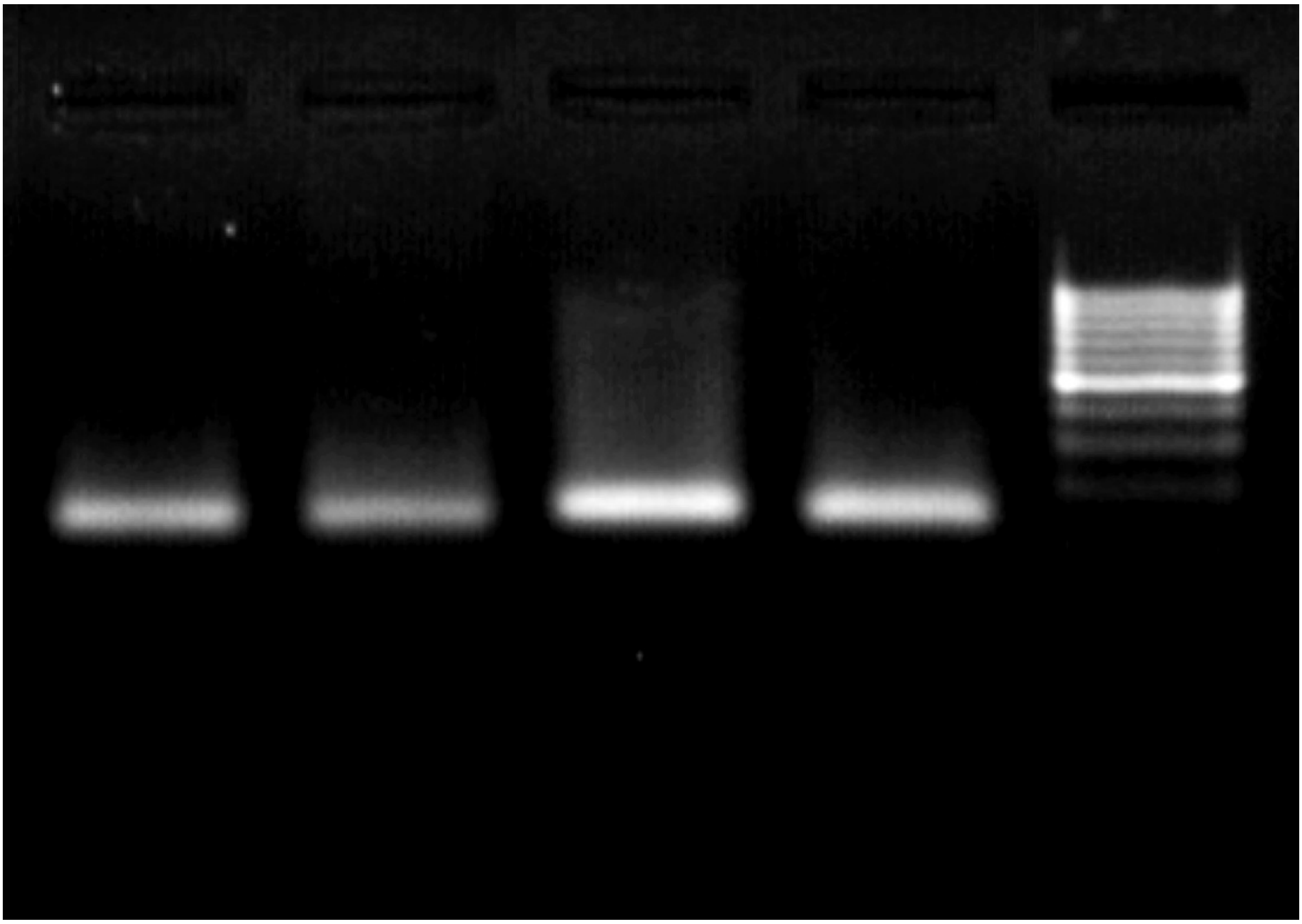

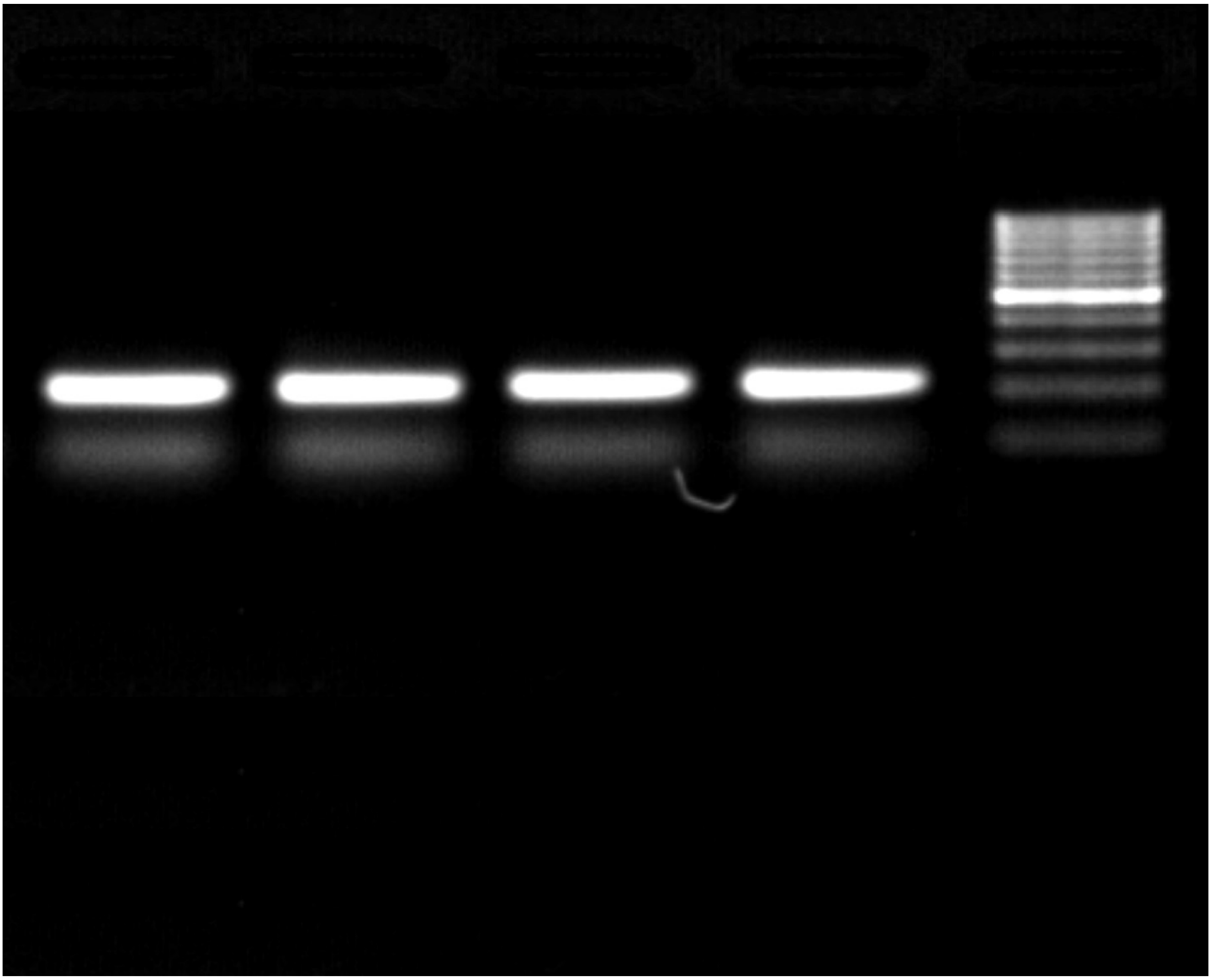

Figure S2: Unlabelled images of histology depicted in Figure 9

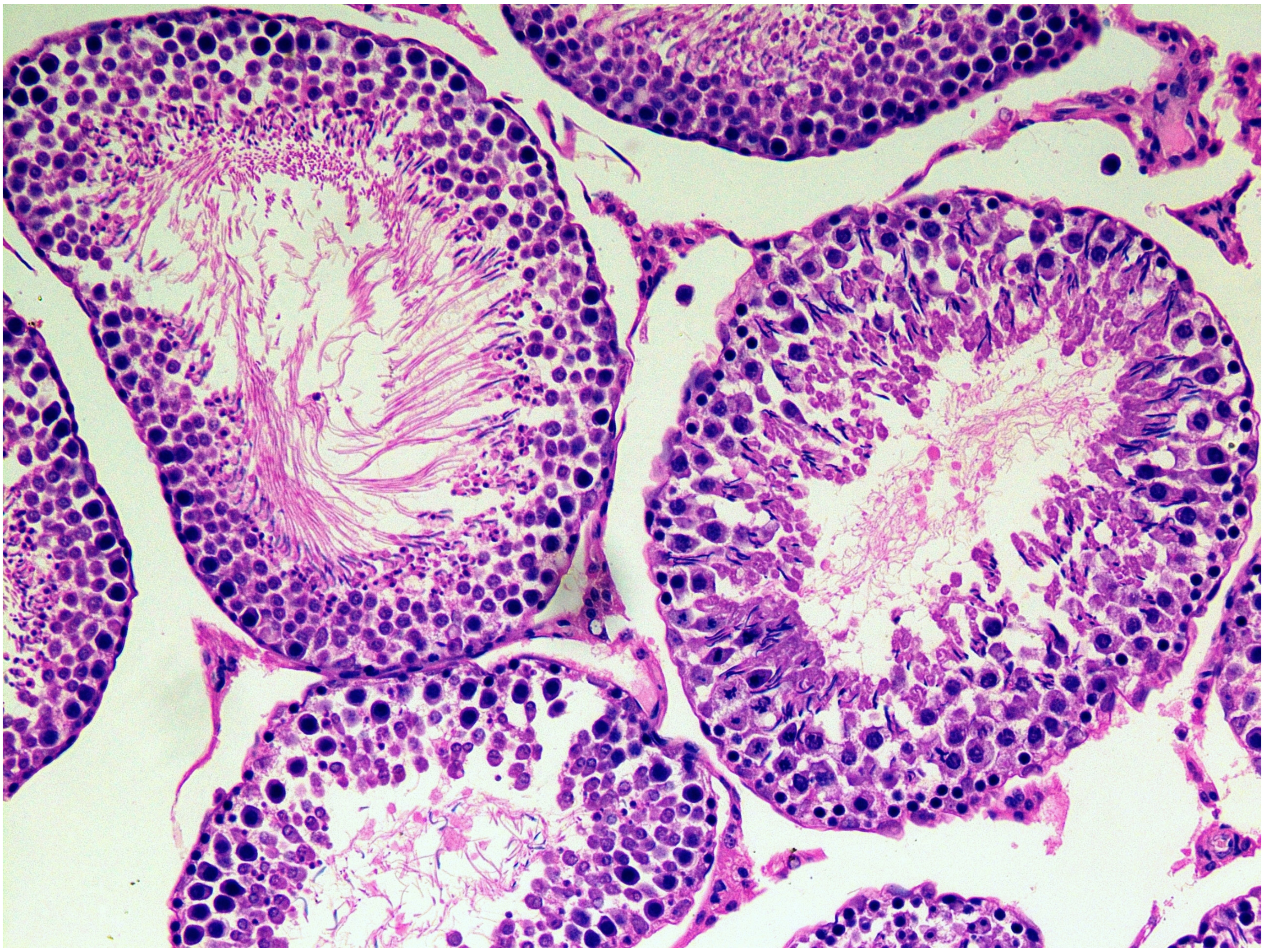

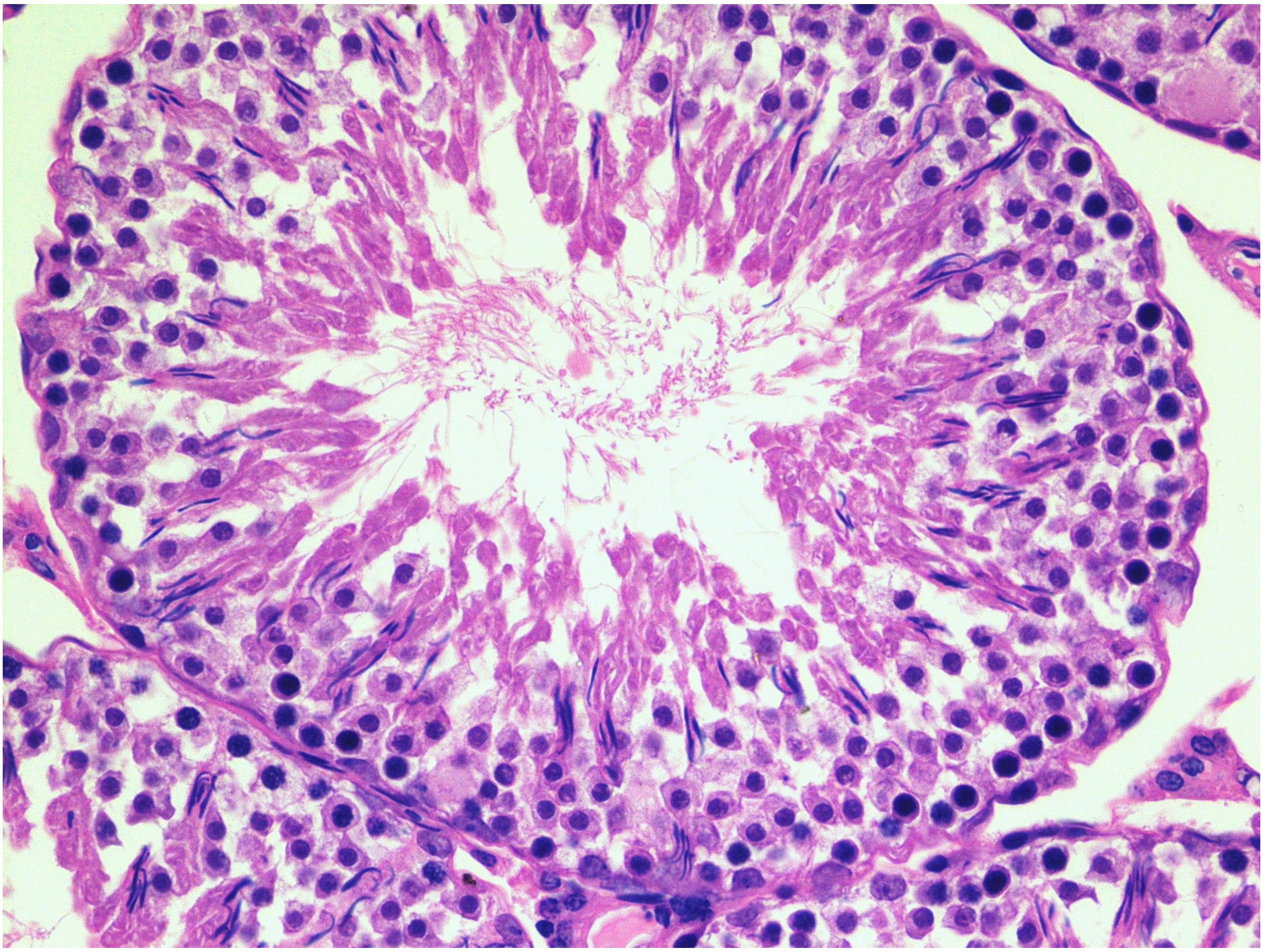

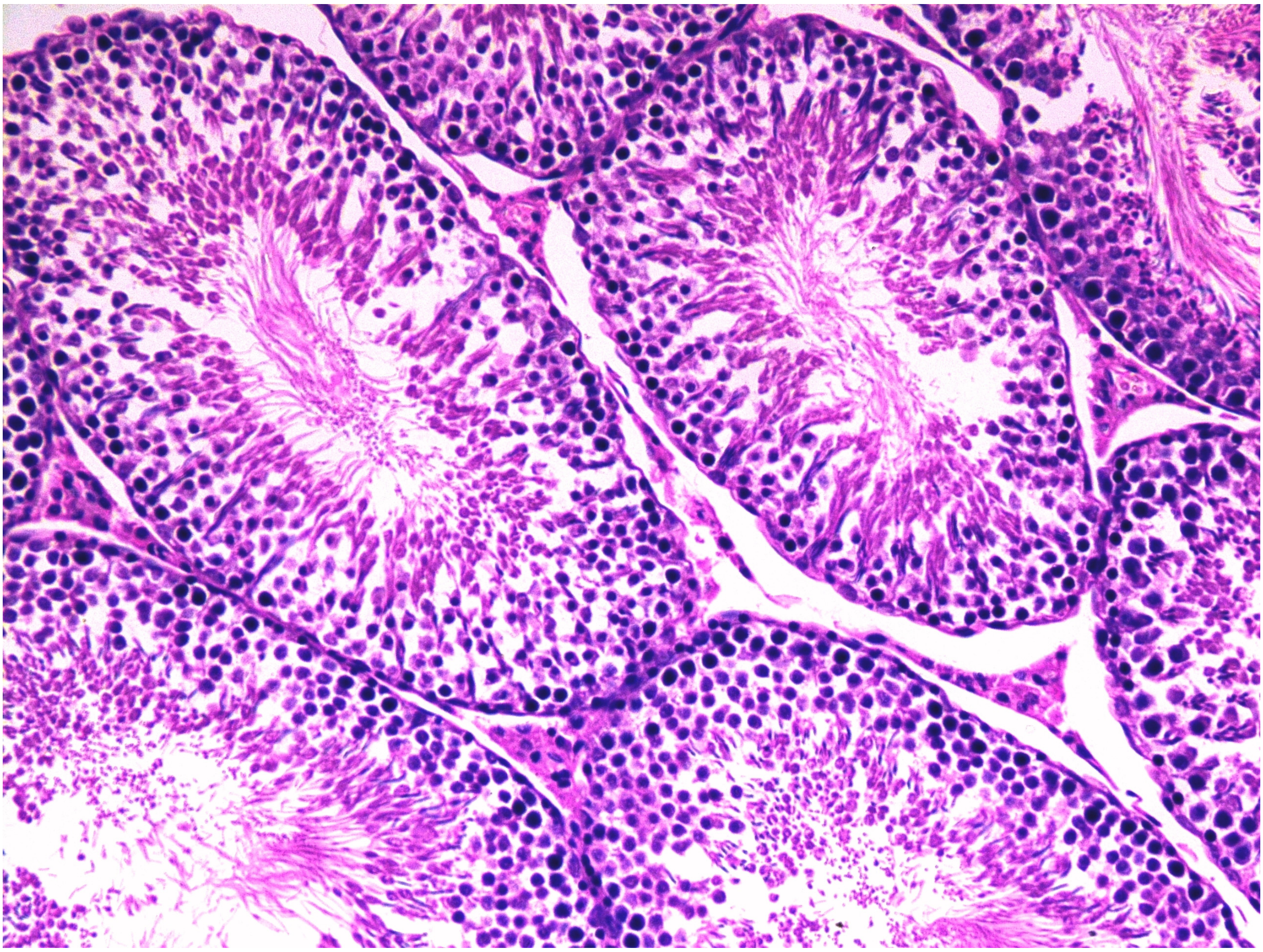

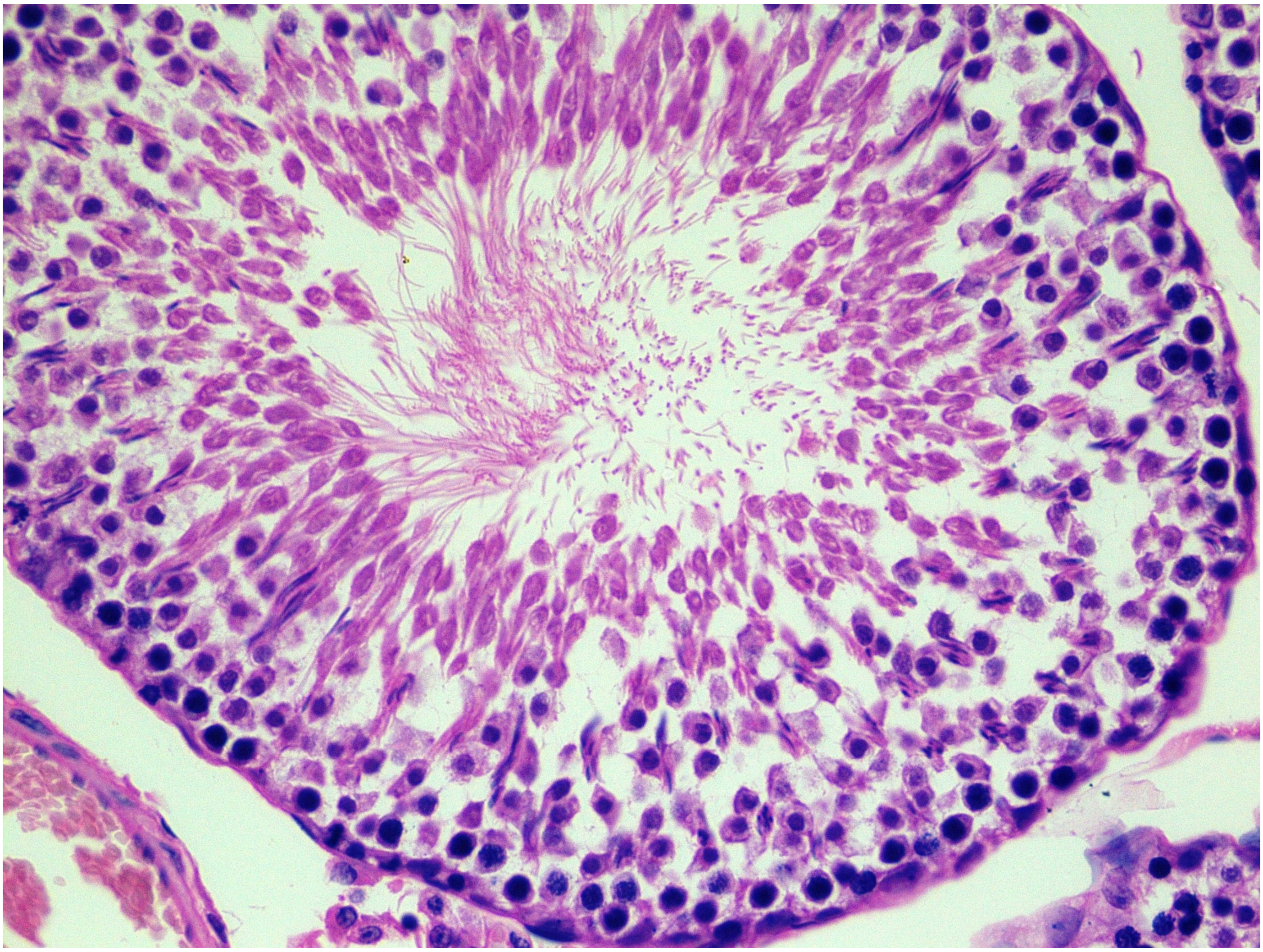

Figure S3: Unlabelled images of histology depicted in Figure 10

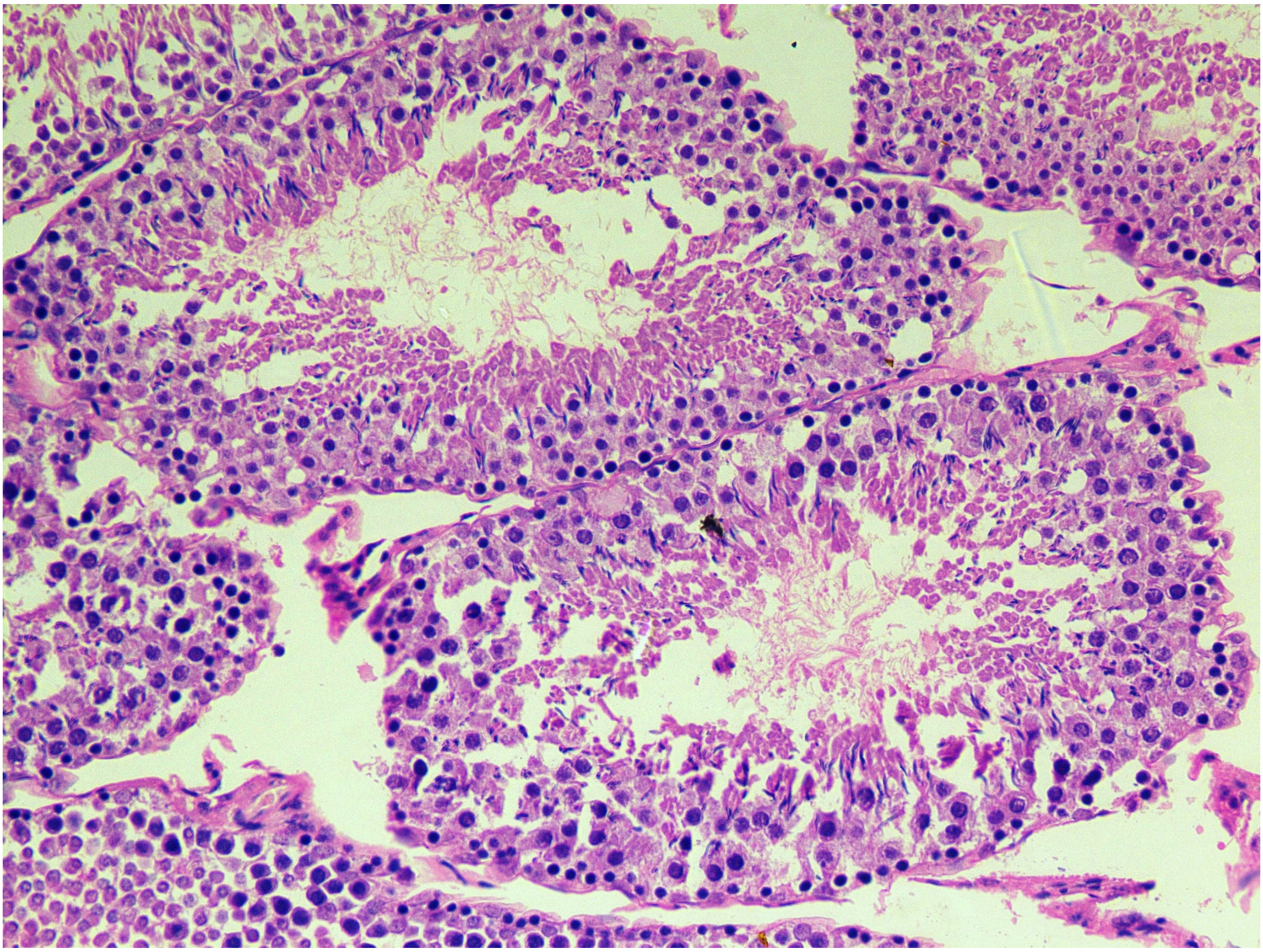

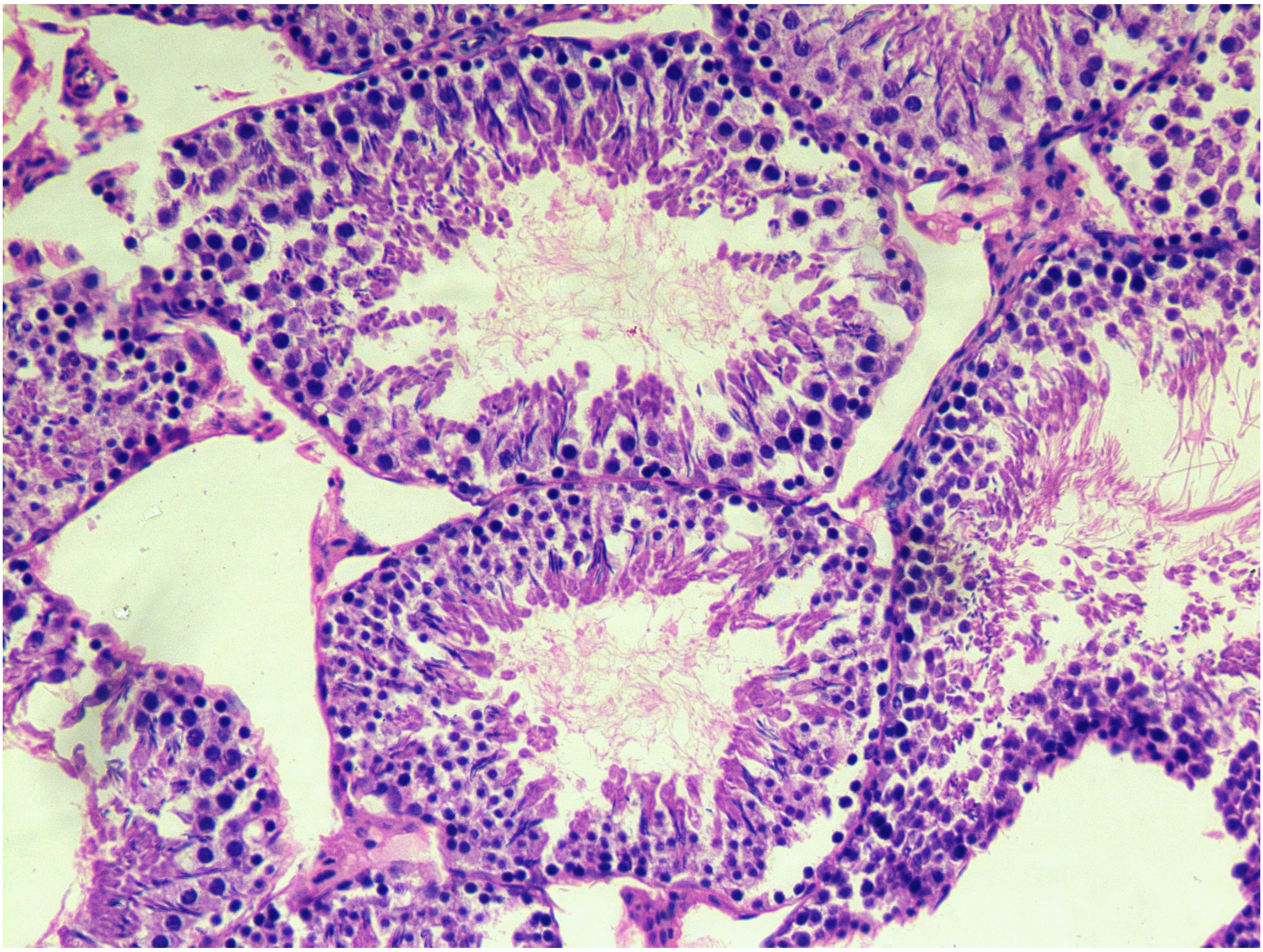

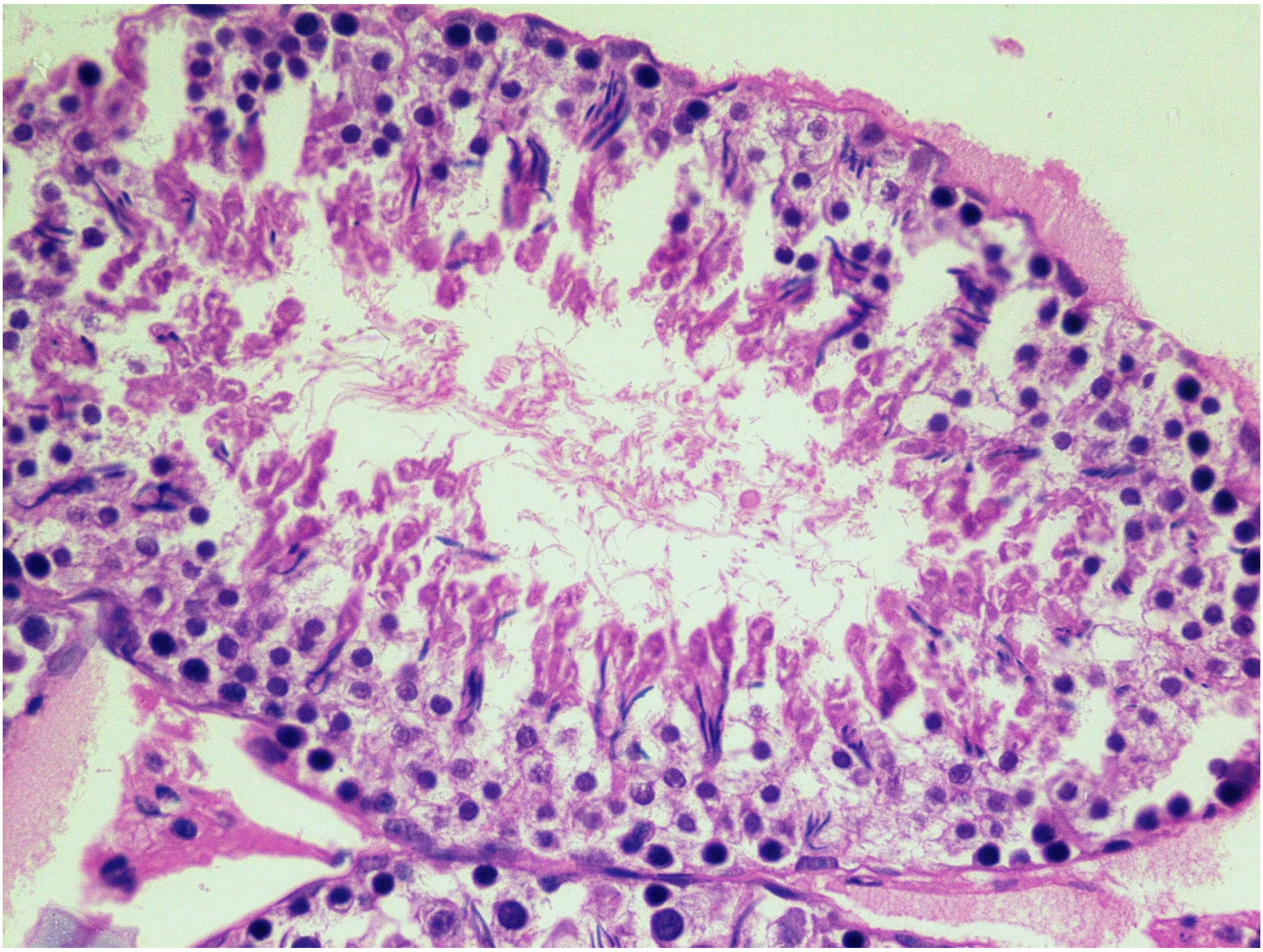

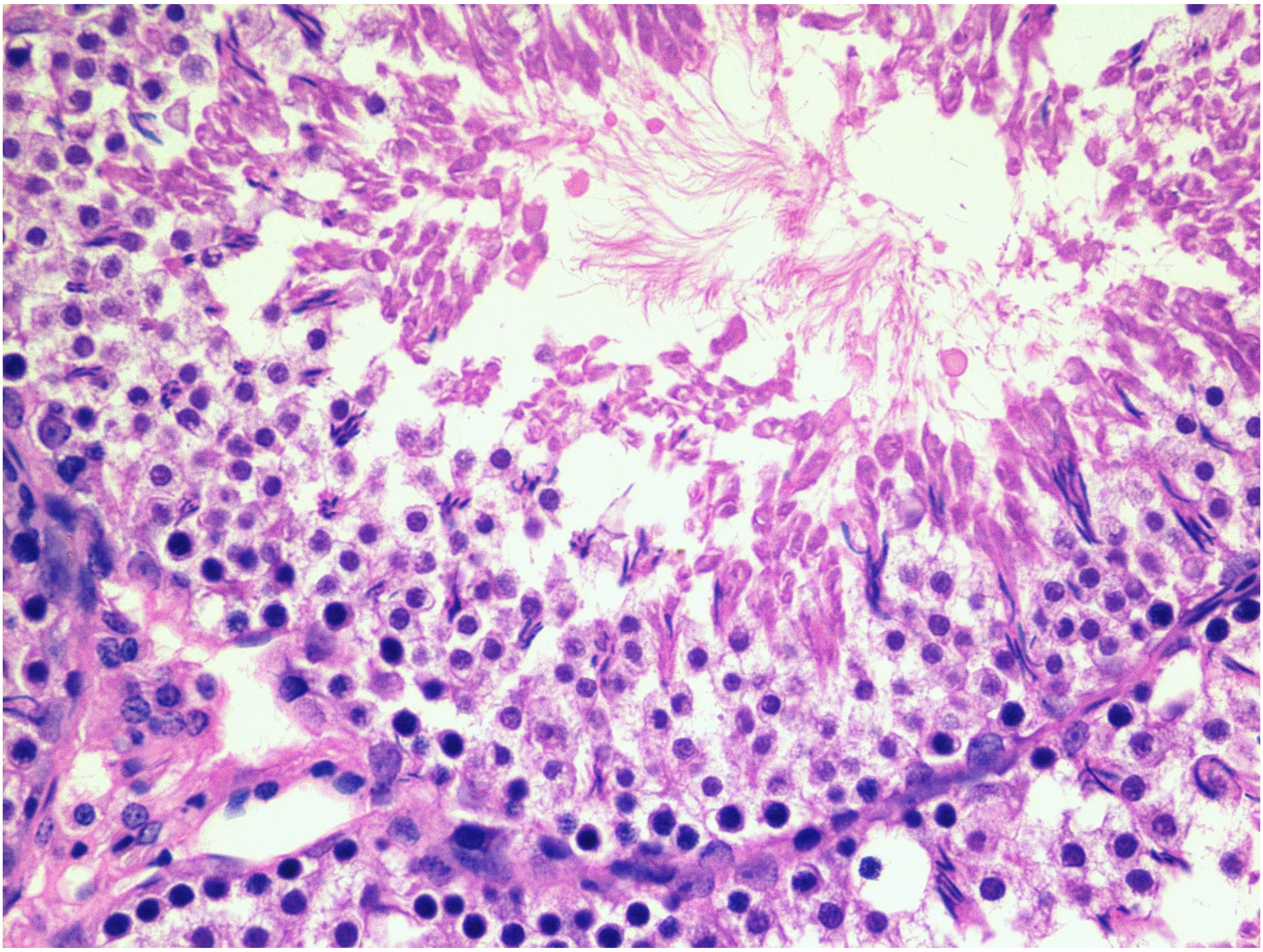

Figure S4: Unlabelled images of histology depicted in Figure 11

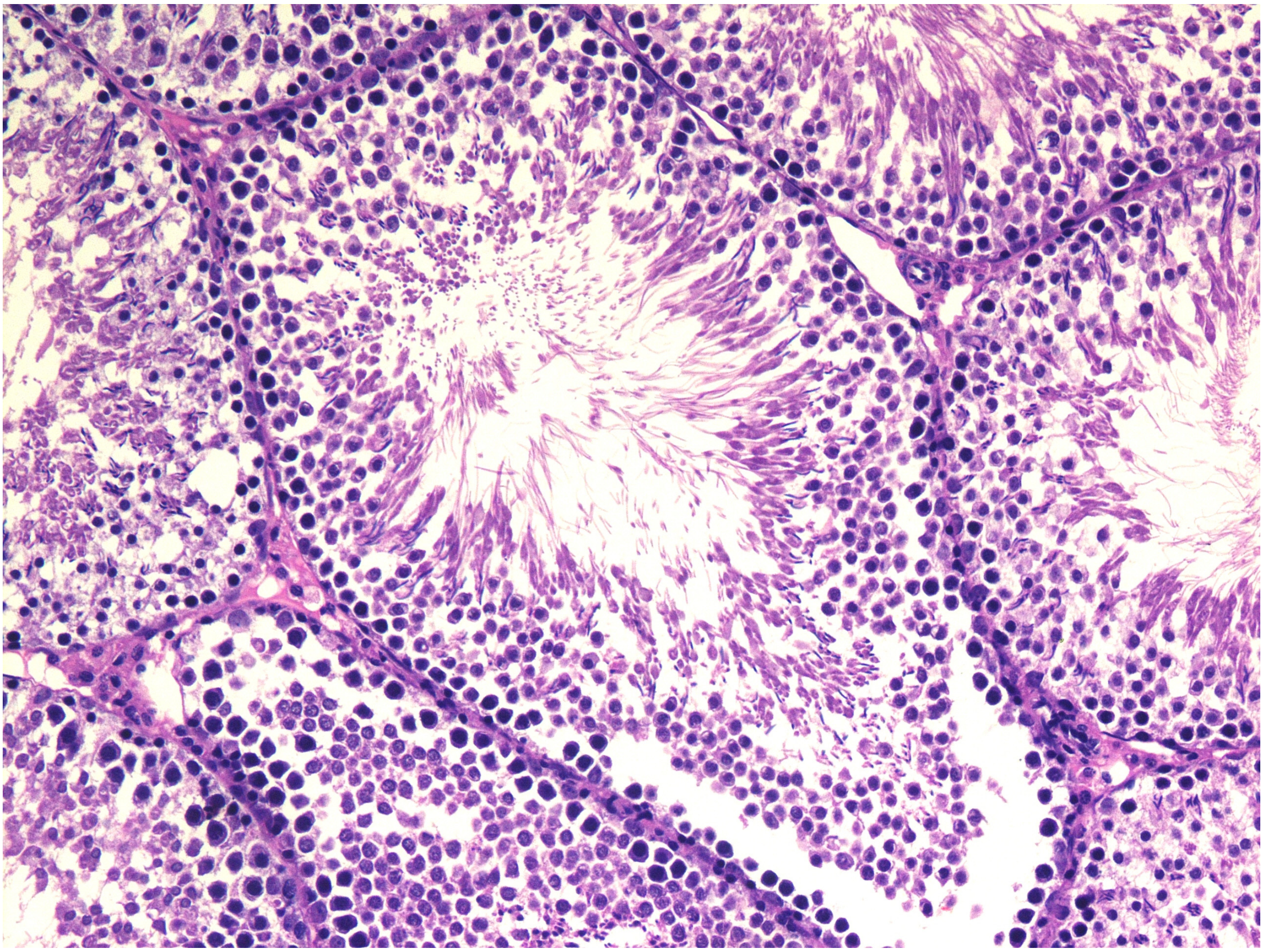

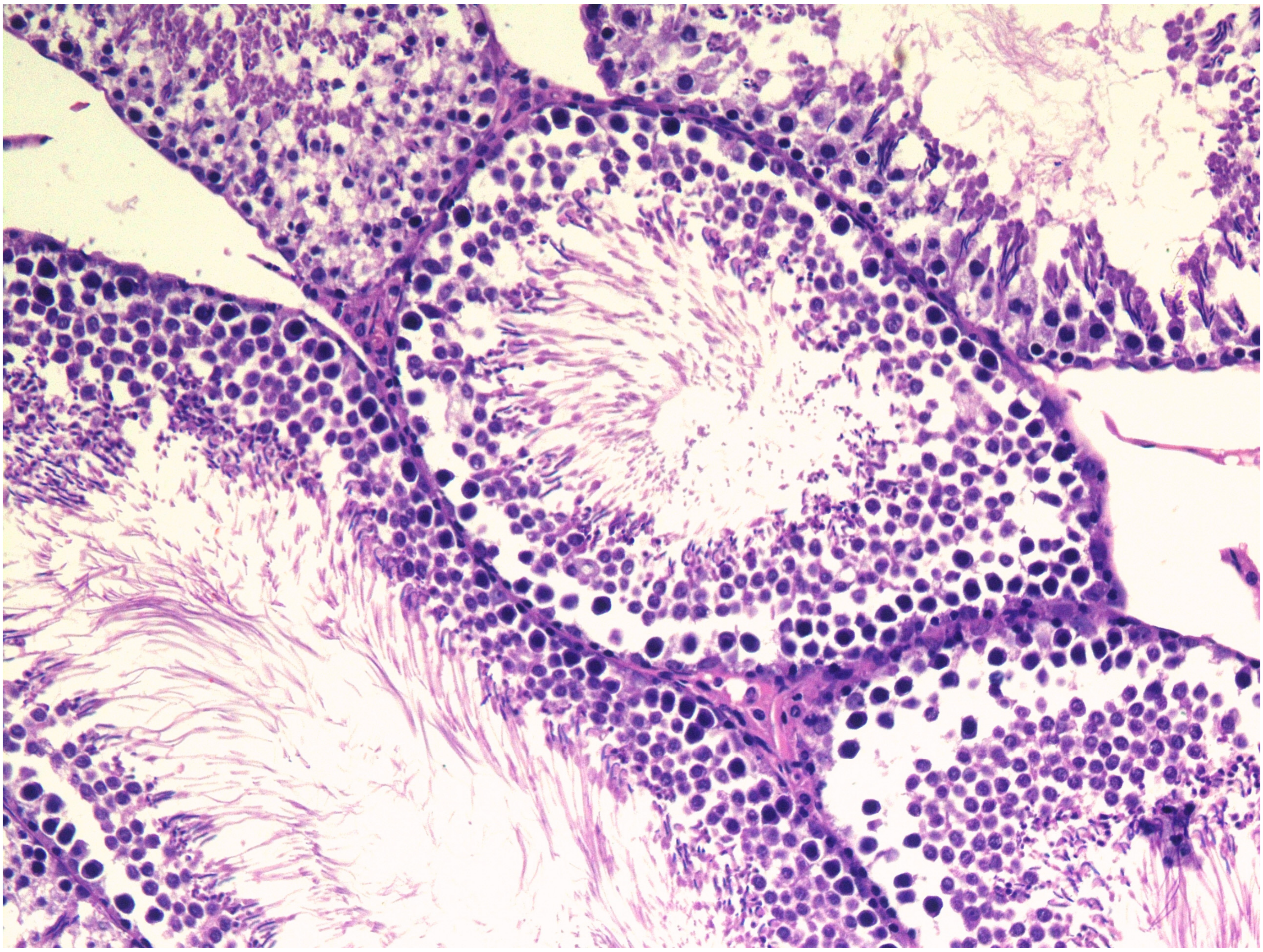

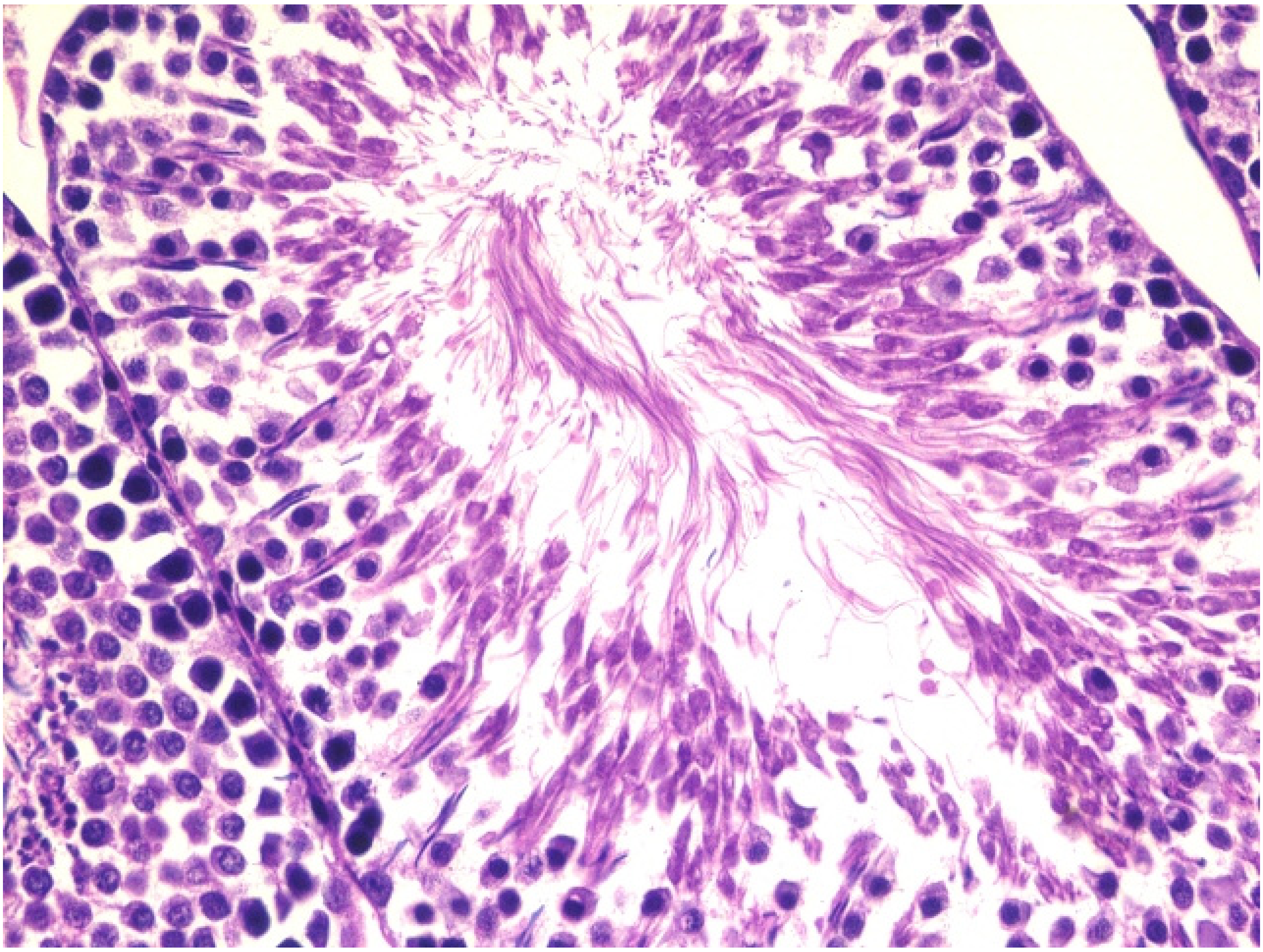

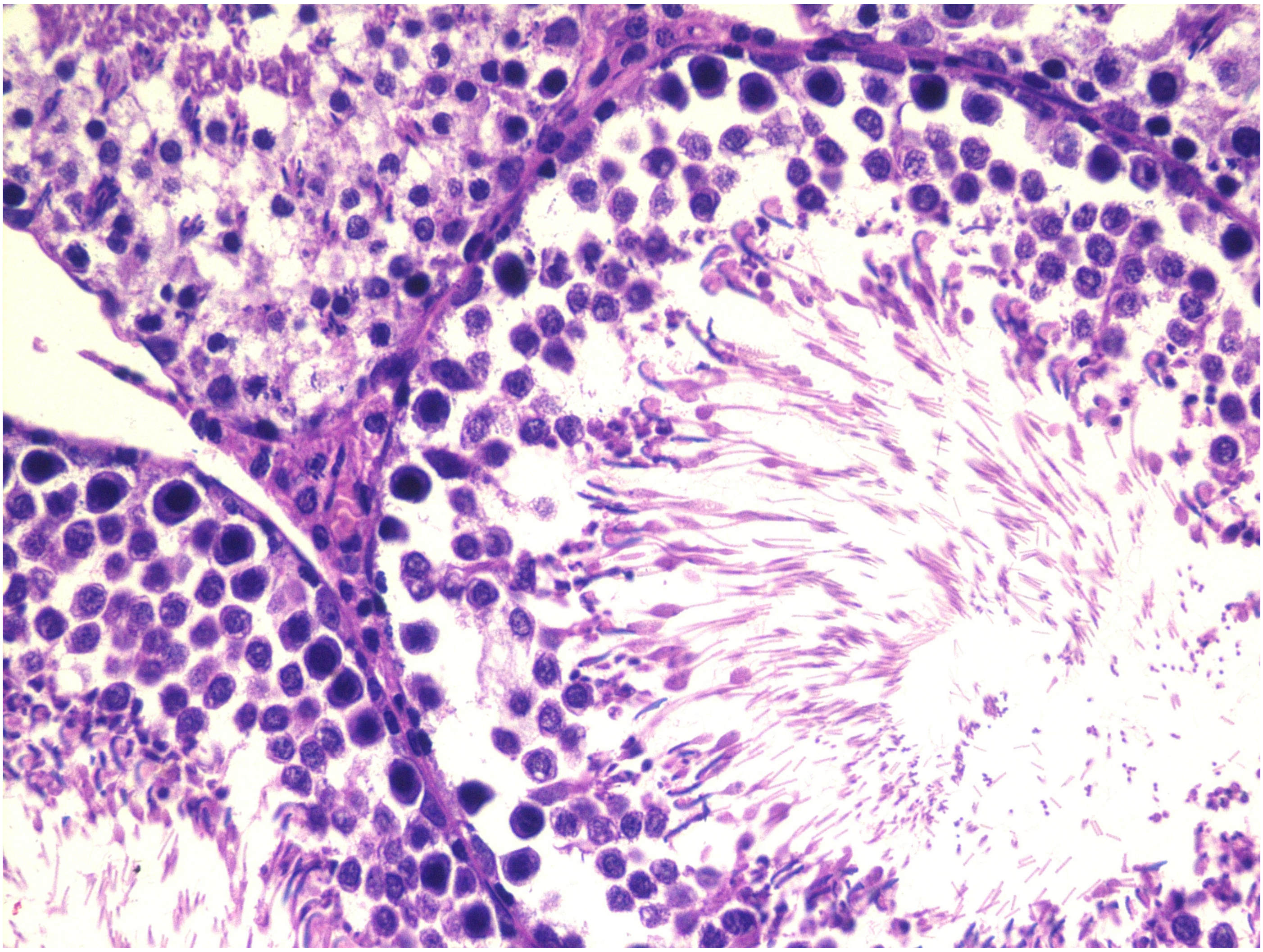

...
